# International comparison of cross-disciplinary integration in industry 4.0: A co-authorship analysis using academic literature databases

**DOI:** 10.1371/journal.pone.0275306

**Published:** 2022-10-17

**Authors:** Yuji Mizukami, Junji Nakano

**Affiliations:** 1 Department of Industrial Engineering and Management, College of Industrial Technology, Nihon University, Narashino City, Japan; 2 Department of Global Management, Faculty of Global Management, Chuo University, Hachioji, Japan; King Abdulaziz University, SAUDI ARABIA

## Abstract

In innovation strategy, a type of Schumpeterian competitive strategy in business administration, "intra-individual diversity" has attracted attention as one factor for creating innovation. In this study, we redefine "framework for identifying researchers’ areas of expertise" as "a framework for quantifying intra-individual diversity among researchers. Note that diversity here refers to authorship of articles in multiple research fields. The application of this framework then made it possible to visualize organizational diversity by accumulating the intra-individual diversity of researchers and to discuss the innovation strategy of the organization. The analysis in this study discusses how countries are promoting research on the topics of artificial intelligence (AI), big data, and Internet of Things (IoT) technologies, which are at the core of Industry 4.0, from an innovation perspective. Note that Industry 4.0 is a technological framework that aims to “improve the efficiency of all social systems,” “create new industries,” and “increase intellectual productivity.” For the analysis, we used 19-year bibliographic data (2000–2018) from the top 20 countries in terms of the number of papers in AI, big data, and IoT technologies. As the results, this study classified the styles of cross-disciplinary fusion into four patterns in AI and three patterns in big data. This study did not consider the results in IoT because of only small differences between countries. Furthermore, regional differences in the style of cross-disciplinary fusion were also observed, and the global innovation patterns in Industry 4.0 were classified into seven categories. In Europe and North America, the cross-disciplinary integration style was similar to that between the United States, Germany, the Netherlands, Spain, England, Italy, Canada, and France. In Asia, the cross-disciplinary fusion style was similar between China, Japan, and South Korea.

## Introduction

In 2011, German Academy of Technical Sciences and German Federal Ministry of Education and Science published the technological framework for Industry 4.0, which was finalized in 2013. Industry 4.0 aims to “improve the efficiency of all social systems,” “create new industries,” and “increase intellectual productivity,” specifically by promoting the use of advanced factories (smart factories) based on a cyber–physical system to improve the efficiency of factory production activities [1]. However, Industry 4.0 is characterized by “predictive maintenance,” which entails anticipating and preventing equipment failures and abnormalities. Thus, studies have focused on artificial intelligence (AI), big data, and Internet of Things (IoT) to create these features [2–4].

With the promulgation of Industry 4.0, the focus of the present study, as well as other scholars, is AI, big data, and IoT technologies, which constitute the core of Industry 4.0. This study examines and discusses each country’s research promotion strategies from the perspective of inducing innovation by integrating different fields.

March [5] defined "Exploration of knowledge" as the process of recognizing distant knowledge (new knowledge) and linking it with existing knowledge. It is the process of accumulating seeds of innovation (already known) for future consideration by the organization. From our perspective, analyzing "interdisciplinary collaborations" helps measure the "exploration of knowledge" of innovation strategies.

This study analyzes the "intrapersonal diversity" of Schumpeterian competition, which is an innovation strategy in three major types of interorganizational competition by Barney [6]. Mizukami et al. [7] observed that researchers with high intrapersonal diversity are more likely to be involved in multiple research areas, such as basic and applied research, while researchers with low intrapersonal diversity are more likely to pursue just one research field. Furthermore, Mizukami et al. [7] found that the accumulation of constituents’ intrapersonal diversity reveals the research fields in which the organization is involved and their connections, allowing for an assessment of the organization’s innovation strategy competitiveness.

Using Mizukami et al.’s [7] analytical framework, this analysis first defines the field of study and the research areas of researchers working in that field. For example, we collect articles in AI-related fields, identify their authors, and objectively derive their areas of expertise and application as well as the connections between these fields. We can then evaluate the competitive strength of an organization’s innovation strategy by overlaying its researchers’ expertise and application fields and their interrelations and deriving their link to the organization’s research areas. In the field of AI, identify which countries (or organizations) researchers are applying AI technology to which research fields. However, this analytical framework remains qualitative.

Next, we adopt Mizukami and Nakano’s [8] analytical framework to quantitatively compare the research field connections, that is, via hierarchical clustering and principal component analyses. Subsequently, we use the method for displaying cross-disciplinary collaboration, which is newly defined in this study, to show the connection patterns of research fields in a network graph. We apply these analyses to AI, big data, and IoT technologies.

For the study data, 19-year bibliographic data (2000–2018) from the top 20 countries in terms of the number of papers in AI, big data, and IoT technologies were included in the analysis.

## Industry 4.0 and its elemental technologies

### Elemental technologies AI, big data, IoT technologies

The smart factory concept of Industry 4.0 connects all machinery, equipment, and management systems in a factory to the Internet, thereby facilitating the manufacturing process and efficiently producing small quantities of a wide variety of high value-added products on a large scale. The networking of the engineering chain and supply chain using IoT, big data, AI, industrial robots, and the Internet, is essential for realizing the smart factory [1].

This study focuses on AI, big data, and IoT technologies, which constitute the core of Industry 4.0. Industry 4.0 proposes a cyber–physical system to realize the smart factory concept. In the physical space of the cyber–physical system, all machinery, equipment, and management systems in the factory are connected to the Internet to collect information. IoT technology is used for information collection. IoT technology is a general term for technologies in which embedded sensors in devices transmit information about the devices via the Internet. The collected information is then stored and processed in cyber space. The big data technology is an umbrella term for technologies that organize and store large amounts of data collected using IoT technology. Finally, “AI technology” refers to advanced technology that examines large volumes of information accumulated via big data [2–4].

### Trends in the number of papers on AI, big data, and IoT technologies

We used the bibliographic data of Web of Science (WoS) core collection, the one the biggest bibliographic database from 2000 to 2018. Our research activities are funded by the Institute of Statistical Mathematics’ Joint Research Program (2019-ISMCRP-1026, 2021-ISMCRP-2036). We have permission to use the Web of Science (WoS) Core Collection, a set of which Clarivate Analytical Inc. provided to the Institute of Statistical Mathematics. This database has been optimized for bibliometric data analysis; using them, some unavailable items on the regular WoS site are accessible for analysis. However, due to contractual regulations, this database only contains data up to 2018 that we have used in our research. Hence, we were easily able to and collect data up to 2018 with relative ease. Conversely, manually extracting data after 2018 from the WoS website is work-intensive, and data preparation is thus, expected to take a long time. Therefore, the study period covered up to 2018.

For the search query for AI-related articles, we set the topic as (TS = "artificial intelligence"). We also set the text type to (DT = = ("ARTICLE" OR "REVIEW")) and the language to (LA = = ("ENGLISH")). Then (PY = = ("<Year>")) was added to specify the year and (CU = = ("Country")) to specify the country. For example, a query to search for IoT-related papers in China in 2018 would be ((TS = "IoT" OR TS = "Internet of Things") AND (DT = = ("ARTICLE" OR "REVIEW") AND LA = = ("ENGLISH")) AND (PY = = ("2018") AND CU = = ("PEOPLES R CHINA")). Similarly, to search for papers related to big data, we changed only the topic to (TS = "Big data" OR TS = "BigData"). In addition, when searching for IoT-related papers, only the topic was changed to (TS = "IoT" OR TS = "Internet of Things").

Topic search is used to search for articles, and the subfields for identifying articles are "Accession Number," "Document Type," "Year Published", and "Language". The subfields for identifying authors are "Author Full Name," "Author Address," "Reprint Author Address", and "Web of Science Categories".

**Fig 1:**
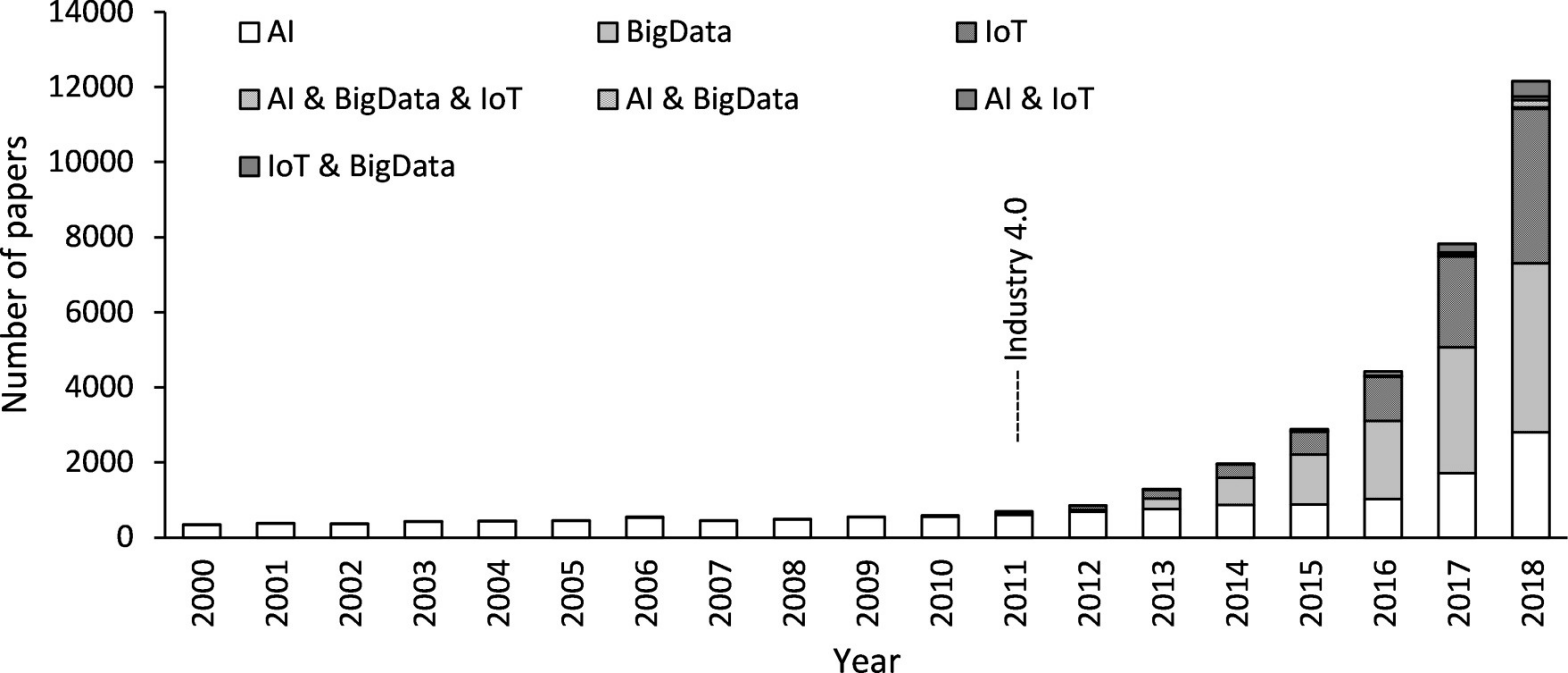
Number of papers on artificial intelligence, big data, and internet of things.

Fig 1 shows the annual change in the number of published papers on these technologies. Studies on AI, big data, and IoT have been continuously increasing, for example, reaching 3,133, 5,155, and 4,662 related papers in 2018, respectively. In 2000, 329 papers on AI were published, which is the largest number of the three technologies. AI is currently under a third boom, with studies accumulating since the 1980s and increasing after 2000. Moreover, the number of papers on big data and IoT has changed since the announcement of Industry 4.0. Before 2011, big data and IoT studies were limited to a few dozen each year. However, after Industry 4.0 was launched, big data and IoT papers have increased rapidly, especially since 2012. Since 2012, several papers related to AI, big data, and IoT have emerged. In 2018, a total of 44 papers were related to all three disciplines—190 to AI and big data, 92 to AI and IoT, and 419 to IoT and big data.

In this paper, the country/region designation of the article is the country/region of the reprint author. And the designation of the country/region of each author is made using the individual address.

Table 1 shows how AI, big data, and IoT papers are ranked by country/region. China and the United States produced the highest number of papers in these fields. In addition, some countries/regions specialize in certain fields. For instance, England is ranked third in AI and IoT; South Korea is ranked third in IoT; India is fourth in AI, big data, and IoT; and Japan is ranked 12th, 13th, and 9th in AI, big data, and IoT, respectively. For country selection, the address in the “Reprint Author Address” field in the WoS was used to eliminate the overlaps. However, in cases where the field contains multiple authors, we used the address of the first author listed.

**Table 1:** Top 20 countries for related papers on artificial intelligence, big data, and internet of things (2018).

	Category											
Rank	All items			AI			Big Data			IoT		
	Country/Region	Papers		Country/Region	Papers		Country/Region	Papers		Country/Region	Papers	
		#	(%)		#	(%)		#	(%)		#	(%)
1	China	2718	23.0	China	509	17.9	China	1293	25.3	China	1080	23.2
2	USA	1865	15.7	USA	500	17.6	USA	1000	19.5	USA	480	10.3
3	KOR	679	5.7	England	152	5.3	England	266	5.2	KOR	446	9.6
4	India	603	5.1	India	152	5.3	India	205	4.0	India	289	6.2
5	England	534	4.5	Iran	104	3.7	KOR	199	3.9	Italy	221	4.7
6	Italy	448	3.8	KOR	101	3.6	Italy	183	3.6	Spain	173	3.7
7	Spain	403	3.4	Australia	89	3.1	Spain	167	3.3	England	162	3.5
8	Australia	314	2.7	Spain	87	3.1	Australia	147	2.9	Taiwan	138	3.0
9	Germany	280	2.4	Italy	73	2.6	Germany	141	2.8	Japan	129	2.8
10	Japan	261	2.2	Canada	70	2.5	Canada	105	2.1	Australia	109	2.3
11	Taiwan	250	2.1	Germany	69	2.4	Taiwan	85	1.7	Canada	92	2.0
12	Canada	246	2.1	Japan	68	2.4	France	84	1.6	Germany	92	2.0
13	Iran	204	1.7	Brazil	63	2.2	Japan	80	1.6	Pakistan	74	1.6
14	France	187	1.6	Turkey	52	1.8	NLD	77	1.5	SAU	74	1.6
15	Brazil	185	1.6	Taiwan	42	1.5	Brazil	60	1.2	Brazil	74	1.6
16	SAU	137	1.2	France	41	1.4	Iran	57	1.1	France	71	1.5
17	Malaysia	127	1.1	Malaysia	39	1.4	Malaysia	46	0.9	Finland	55	1.2
18	Turkey	123	1.0	Poland	36	1.3	CHE	45	0.9	Belgium	48	1.0
19	NLD	122	1.0	Russia	35	1.2	Poland	42	0.8	Iran	48	1.0
20	Pakistan	116	1.0	SAU	35	1.2	SAU	38	0.7	Malaysia	46	1.0
After 20th		2041	17.2		526	18.5		797	15.6		760	16.3
Total		11843	100.0		2843	100.0		5117	100.0		4661	100.0
United Kingdom of Great Britain and Northern Ireland												
-	England	534	4.5	England	152	5.3	England	266	5.2	England	162	3.5
-	Scotland	68	0.6	Scotland	16	0.6	Scotland	34	0.7	Scotland	19	0.4
-	Wales	27	0.2	Wales	12	0.4	Wales	11	0.2	Wales	7	0.2
-	NIR	11	0.1	NIR	2	0.1	NIR	4	0.1	NIR	6	0.1
GBR Total		640	5.4		182	6.4		315	6.2		194	4.2

Note: If the country/region name was too long to fit in the cell, a three-digit ISO 3166–1 code was used. These three-digit codes indicate the following countries/regions: CHE (Switzerland), GBR (United Kingdom of Great Britain and Northern Ireland), KOR (South Korea), NIR (Northern Ireland), NLD (Netherlands), SAU (Saudi Arabia), and USA (United States of America).

Note that "England," "Wales," "Scotland," and "Northern Ireland" are treated as separate data. Table 1 also shows that the data for these countries are presented collectively as “GBR,” and there are some changes in the international ranking of “England.” “GBR” moves up one place to sixth in IoT and one place to fourth in the overall international ranking. Besides, "Peoples R China" and "Taiwan" are treated as separate data.

## Review of related areas

### Types of interorganizational competition in business strategy

Barney [6] stated that there are three types of interorganizational competition: industrial organization (IO), Chamberlainian, and Schumpeterian. The analytical approach in this study provides a measurable framework for the concept of intrapersonal diversity in Schumpeterian competition. It also provides an extended framework for evaluating organizations’ competitiveness by gathering information on intrapersonal diversity. In addition, the position of the analytical method in interorganizational competition was defined for the first time in this study. The following sections describe the characteristics of the three types of interorganizational competition to illustrate the position of the analytical method used in this study.

IO competition is based on the structure–conduct–performance (SCP) model of industrial organization theory in economics, which was proposed by Mason [9] and systematized by Bain [10]. The SCP model is used to discuss how industrial structure, corporate behavior, and performance are linked, and Porter [11] applied this idea to business strategy. Porter [11] stated that in IO competition, the more the competitive environment of an industry or an industry deviates from perfect competition and approaches perfect monopoly, the more profitable the companies in it will be. Porter [11] also stated that business strategies should be developed to position a company within an industry or industry such that to avoid severe competition with rivals, build barriers to entry for new companies to enter market, and promote differentiation by enhancing brand power.

Next, Chamberlainian competition is based on the resource-based view (RBV) systematized by Barney [12]. RBV is a model that states that to achieve “winning differentiation,” it is important to use the organization’s internal resources, such as technology, knowledge, brand, and human resources. Both IO and Chamberlainian competition explain an organization’s strategy and performance, but IO competition focuses on the positioning of an organization within an industry or industry, while Chamberlainian competition focuses on the using resources within the organization. The transition of RBV formation began with Chamberlin’s point that “the source of an organization’s competitiveness lies in the utilization of the company’s internal resources, such as its technology, knowledge, brand, and human resources [13].” Later, Penrose [14] stated that “organizations grow by learning how to utilize resources such as human resources and technology through experience.” Then, Wernerfelt [15] used the term RBV for the first time, stating that “organizations can increase their excess profits by monopolizing resources.” Furthermore, unlike Wernerfelt [15], Barney [6] showed that organizational resources are more important in RBV.

Dierickx and Cool [16] stated that, in Chamberlainian competition, "Even if an organization can monopolize a resource temporarily, its value will not last long if it is imitated by others. The resource must be difficult for others to imitate.” Thus, while Wernerfelt [15] and Barney [6] focused on the “monopoly of resources,” Dierickx and Cool [16] focused on the “difficulty of imitation of resources.” Porter’s [17] activity system is an actual framework that realizes the difficulty of imitating resources. In the activity system, organizational behaviors (activities) are closely related to each other, and these relationships are the source of organizational differentiation.

Finally, Schumpeterian competition is a strategy based on innovation. Innovation is a method for thinking to create new knowledge (value) from “new combinations of knowledge, resources, and experience in economic activities,” which Schumpeter [18] named new combination. March [5] categorizes the innovation process into "exploration of knowledge" and "exploitation of knowledge." "Exploration of knowledge" is the process of recognizing distant knowledge (new knowledge) and linking it with the existing, and it is the process of accumulating the existing seeds of innovation for future consideration by the organization. "Exploitation of knowledge" refers to activities that deepen knowledge by combining nearby "already known" knowledge. It is a process by which an organization can earn profits.

Both IO and Chamberlain competitions share a common characteristic, in that both are suited to industries in stable business environment and reasonably foreseeable future is. Companies can plan and strategize in a slowly changing environment and a fairly predictable future. However, the current business environment may differ. Globalization, deregulation, and, above all, the rapid development and digitization of IT have accelerated change in the business environment. Thus, in these industries, predicting the future is becoming impossible. D’aveni [19] referred to this environment as "hyper competition. Alternatively, the type of competition is adapting to the Schumpeterian competition.

### Exploration and Exploitation of knowledge in Schumpeterian competition

In Schumpeterian competition, March [5] defined ambidexterity as the importance of a considerably high balance between the activities of "exploration of knowledge" and "exploitation of knowledge" for an organization’s long-term growth. However, the organization is often biased toward "Exploitation of knowledge" activities and not "Exploration of knowledge" activities, resulting in the exhaustion of ideas. This phenomenon is defined as a "Competency trap." The difference in the characteristics of "Exploration of knowledge" and "Exploitation of knowledge" is cited as a factor that leads to an organization falling in to a state of competency trap. As "Exploration of knowledge" is an activity to recognize distant knowledge (new knowledge), acquiring these values systematically is challenging and involves risks. However, "Exploitation of knowledge" is an activity to combine existing knowledge, so the prospect is more certain and it is easier to plan the activity. When organizations seek short-term efficiency, they can benefit from proactively working on "Exploitation of knowledge." However, from a medium- to long-term perspective, March [5] stated that without "exploration of knowledge," ideas will eventually dry up and innovation-type growth will slow down.

Organizations try to avoid falling into a competency trap by activating "exploration of knowledge." First, there is Chesbrough’s [20] Open Innovation, which seeks the source of knowledge from outside, and Rothaermel and Alexandre [21] as an empirical study, who conducted a questionnaire survey on 4195 employees of 41 business units of 10 multinational companies. The survey investigated whether they outsourced (procured externally) or produced internally using existing technologies while acquiring new technologies. The survey results showed that companies that used a balanced both in-house production and outsourcing when acquiring new technologies had higher ex-post return on equity (ROE) and the number of patents acquired.

"Diversity of organizational members" is a mechanism inside an organization to avoid falling into a competency trap. Beckman [22] conducted an empirical study involving a combination of interviews, questionnaires, and archival research with 141 Silicon Valley companies in the fields of hardware and software, telecommunications, medicine, and biotechnology. In the survey, members of the founding team were asked about their history since the company’s inception. If the words "cutting edge," "pioneer," or "ahead of the curve" were included in the response, the company was judged to have adopted an exploratory strategy. The analysis showed that if the founding team members had previously worked for various startups, they were more likely to adopt an exploratory behavior.

In an empirical study on the effect of intrapersonal diversity on organizational performance, Bunderson and Sutcliffe [23] collected data on the profiles of management members of 44 Fortune 100 companies and found that companies with management members experienced in various functions such as finance, R&D, sales, and marketing (i.e., management members with high intrapersonal diversity) performed better. The study found that companies with management members with experience in various functions, including finance, R&D, sales, and marketing (i.e., management members with high intrapersonal diversity), tended to perform better.

### Bibliometric approach to research evaluation

Obtaining indicators that allow analysis from different perspectives and dimensions (productivity, visibility and influence, and networks) helps to understand the subjects’ behavior and tendencies and provides essential information for the management of scientific activities and aids in the generation of new knowledge by professionalized communities [24].

Using the bibliometric method, researchers can base their work on aggregated bibliographic data created by other scientists expressing their opinions through citations, collaborations, and writings. These data can be aggregated and analyzed to gain insights into the structure, social networks, and topical interests of the field [25]. Bibliometric methods have two main applications: performance analysis and science mapping [26]. Performance analysis assesses individuals’ and institutions’ research and publication performance. Science mapping reveals the structure and dynamics of a scientific field. In this study, science mapping exposes each country’s innovation strategy in the technology field of Industry 4.0 using the information on scientific field obtained from relevant scientists’ papers. Although bibliometric methods are not new [27, 28], they have gained popularity because of the proliferation of easily accessible online databases containing citation data (i.e., Web of Science (WoS)) and the increasing use of the Internet. Zupic and Cater [25] developed the software for performing bibliometric analysis that we have used here.

We discuss five methods for analyzing bibliometric data: the following methods use citation data to construct measures of influence and similarity: citation, co-citation, and bibliometric coupling; followed by co-authorship analysis, which uses co-authorship data to measure collaboration; and finally, co-word analysis, which measures the association between concepts that co-occur in a document’s title, keywords, or abstract.

Citation analysis shows the number of studies, authors, or journals cited in the study area. The number of citations is used as a measure of influence; the higher the number of citation the higher the importance. While citation analysis provides information about the relative influence of publications, it lacks the ability to identify the network of interconnections among scholars [29].

The underlying assumption of co-citation analysis [28, 30] is that the more two items are cited together, the more likely their content is related. Depending on the unit of analysis, different types of co-citation can be used, such as document, author [31–33], and journal co-citation analyses [34].

Bibliographic coupling analysis [27] uses the number of references shared by two documents as a measure of their similarity. The higher the overlap of the bibliographies of two papers, the stronger their bibliographical coupling. However, it is quite possible for a bad scientific work to receive more citations than a merely mediocre one [35]. However, it is rare for a work to be cited for negative reasons, and scientists generally do not criticize the past literature [36]. In mapping science, co-citation is used more frequently than bibliographic coupling [37].

Co-authorship analysis examines the social networks formed when scientists collaborate on scientific papers [38]. A relationship is established between two authors when they co-publish a paper [39]. Co-authorship in scientific publications is presumed to be an indicator of collaboration. As co-authorship reflects stronger social ties than other relevant indicators, it is suitable for examining social networks rather than the intellectual structure of a research field. Additionally, as bibliographic data include information on authors’ institutional and geographical affiliations, co-authorship analysis can help examine collaboration issues at the institutional and national levels [25].

Co-word analysis [40] is a method for content analysis that uses words in a document to establish relationships and build the conceptual structure of a domain. Frequent co-occurrence of a word in a document means that the concepts behind the word are closely related.

### Interdisciplinary integration in research capabilities

Leydesdorff and Ivanova [41] argued that policymakers often explore the effects of “synergy” when they seek “cross-disciplinary fusion,” because crossing disciplinary boundaries is often needed to address problems. This study discusses recent advances in the application and measurement of “cross-disciplinary fusion” and proposes an information theory-based method for measuring “synergy.”

Such interdisciplinary approach in the field of academic studies include “joint research between different organizations,” “joint research between different research fields,” and “joint research through industry–academia–government collaboration.”

Related studies on “joint research between different organizations” can be found in Mizukami et al. [42], who proposed a method for measuring these collaborations based on paper co-authorship, assuming that joint research within and outside the organization plays an important role in generating innovation to enhance its research capability. This method extends the concept of mediation centrality index of network theory to apply to organization theory, which allows for an aggregation of the connections within, outside, and inside the firm separately and the management of ease of information flow within and outside the organization, aiming at firms that innovate easily. This is an analytical framework for the "boundary spanner" presented by Leifer and Delbecq [43] and Ancona and Caldwell [44], and elaborated by Friedman and Podolny [45] using social network theory.

Mizukami et al. [46] presented another international comparison under “collaboration between different organizations” and calculated the ratios of these mediation types for each individual, aggregated them by country, and expressed the distributions as Lorenz curves [47] and Gini coefficients [48] to examine the relation between mediation types and innovation diffusion. Mizukami et al. [7] conducted another relevant study on “collaboration between different research fields” and proposed co-authorship analysis as method for deriving researchers’ fields of expertise and objectively defining them. This study proposes a method for identifying fields where joint research is actively conducted under “joint research between different research fields.” It provides a measurable framework for the concept of “intrapersonal diversity” in the Schumpeterian competition innovation strategy. Specifically, the researcher’s intrapersonal diversity is captured, accumulated, and evaluated as the organization’s competitiveness. This analysis method is used in the analysis of this paper. The details of the analysis method are presented in the next chapter.

## Analytical methods

### Identifying the researcher’s area of expertise

A researcher’s initial specialization can be judged by the doctoral degree obtained and similar information. For example, in WoS, Table 2 shows 23 research fields, and it is easy to judge them according to this classification. However, in their subsequent research activities, they may have been active in other fields and made academic contributions; for example, it cannot be said that the field of their doctorate coincides with the field in which they have been active and made academic contributions in the past three years. The researcher’s field of expertise was sometimes defined subjectively and sometimes based on each individual’s application. Moreover, such a definition was subjective, at times excluding achievements in these fields, which muddled the objectivity of the researcher’s field of expertise [7].

**Table 2:** Classification of research fields.

#	Subject Area	#	Subject Area
1	Agricultural Sciences	13	Microbiology
2	Biology & Biochemistry	14	Molecular Biology & Genetics
3	Chemistry	15	Multidisciplinary
4	Clinical Medicine	16	Neuroscience & Behavior
5	Computer Science	17	Pharmacology & Toxicology
6	Economics & Business	18	Physics
7	Engineering	19	Plant & Animal Science
8	Environment/Ecology	20	Psychology/Psychiatry
9	Geosciences	21	Social Sciences, general
10	Immunology	22	Space Science
11	Materials Sciences	23	Arts & Humanities
12	Mathematics		

*Note*. Essential Science Indicators Subject Areas in the Web of Science Core Collection

Table 2 shows how the research areas in this study are classified based on the Essential Science Indicators Subject Areas [49] in the Web of Science (WoS) Core Collection. Understand the degree of concentration (or diversity) of researchers in their fields of expertise is also essential. For example, researcher X specializes in a specialized field and is devoted to research in that field. Researcher Y, on the other hand, may be involved in both the specialized field and the applied field where the knowledge is utilized. Thus, there is a need for a balanced distribution of both types of researchers to create and disseminate academic knowledge and contribute to the development of the world [7].

The 23 Essential Science Indicators Subject Areas in the Web of Science Core Collection were used for the article specialties. This data was generated from the "Web of Science Categories” using a conversion table [50].

**Fig 2:**
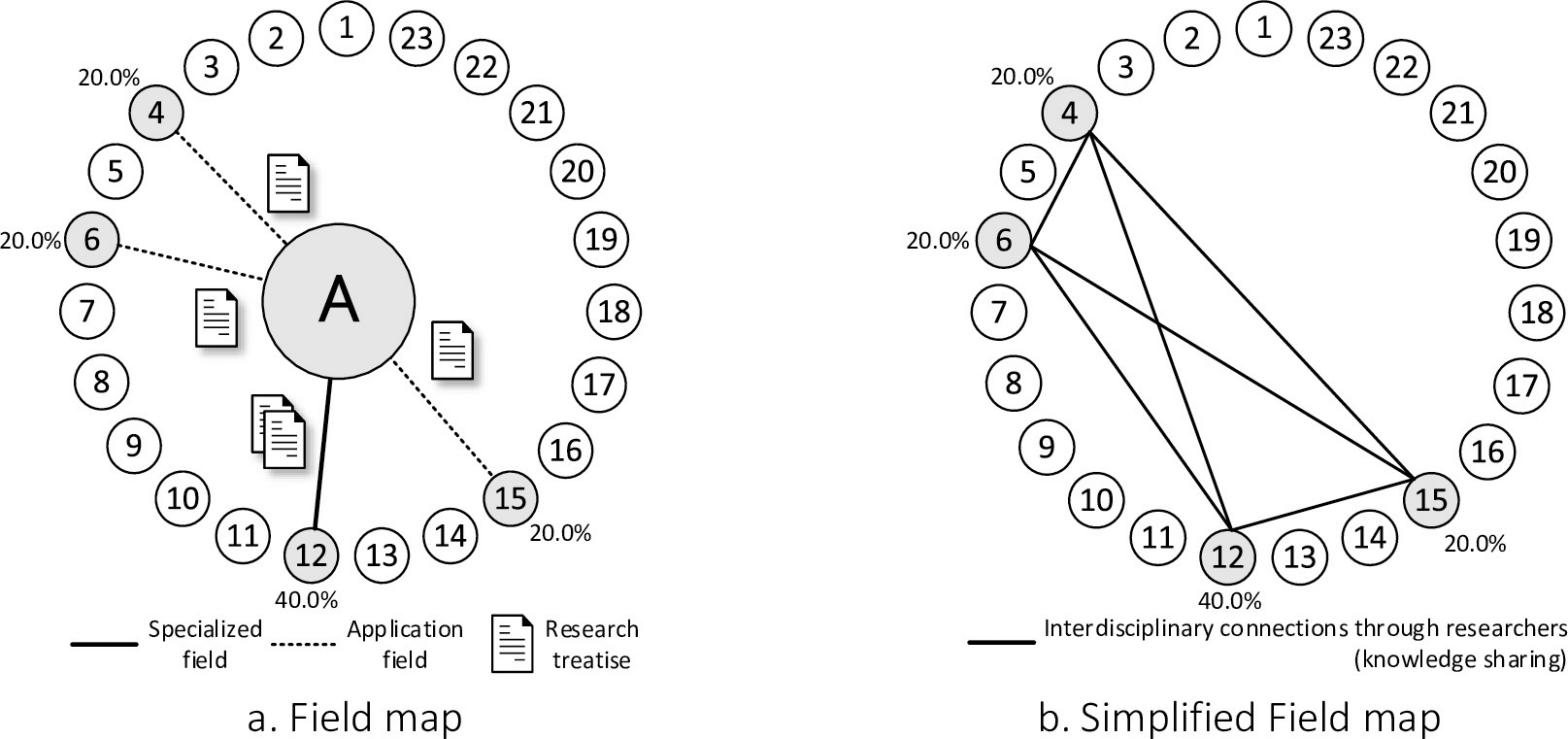
Examples of researcher A’s areas of expertise and their applications.

To address this issue, Mizukami et al. [7] proposed deriving the field of expertise from authorship information for an objective definition. Fig 2(A) shows researcher A’s field of expertise and its applications. If researcher A published two papers in mathematics (12), one in clinical medicine (4), one in economics and business (6), and one in general fields (15), his/her field of specialization is mathematics, with a 40.0% degree of concentration. If the degree of concentration is high, a researcher is considered to focus on research in his/her specialization field. Conversely, if the degree of concentration is low, a researcher is considered to apply research results in the specialized field to other fields.

This study uses WoS as a source of information. In WoS, some journals with all their papers fall under more than one specialization field. The first method is "select the first field;" however, this study does not use this method because it prioritizes papers with smaller numbers in the Essential Science Indicators Subject Areas (ESISA) shown in Table 2. Next is the "random selection" method, which helped eliminate the bias of prioritizing the papers with the lowest ESISA numbers when we tried this method in the WoS, but the authors with few papers showed different results in each analysis. Therefore, this method was excluded in this study. Finally, we used the "split by proportions" method (50.0% for each of the two specified specialties and 33.0% for each of the three specified specialties). When this method was tried in WoS, both the bias of prioritizing papers with the lowest ESISA numbers and the phenomenon of different results for authors with few papers were eliminated.

However, the information of each researcher shown in Fig 2(A) does not show the connection between each research field unless it passes through the researcher located at the center of the figure, and the linkage is unclear. Therefore, in this method, we used a simplified indication method for reconstructing the information about each researcher into the information between the fields. Fig 2(B) shows an example of the simplified indication methods and the connection between each field is clarified.

Researchers who are involved in various research fields are considered to have high intrapersonal diversity (low concentration), and their contribution is considered high from the perspective of Schumpeterian competition in innovation strategy. However, those with low intrapersonal diversity (high concentration) are assumed to be focusing on few research fields and thus considered to have the ability of Chamberlainian competition. However, when evaluated from the Schumpeterian competition perspective, their contribution is small.

### Visualization of the organization’s research capacity and degree of interdisciplinary integration

**Fig 3:**
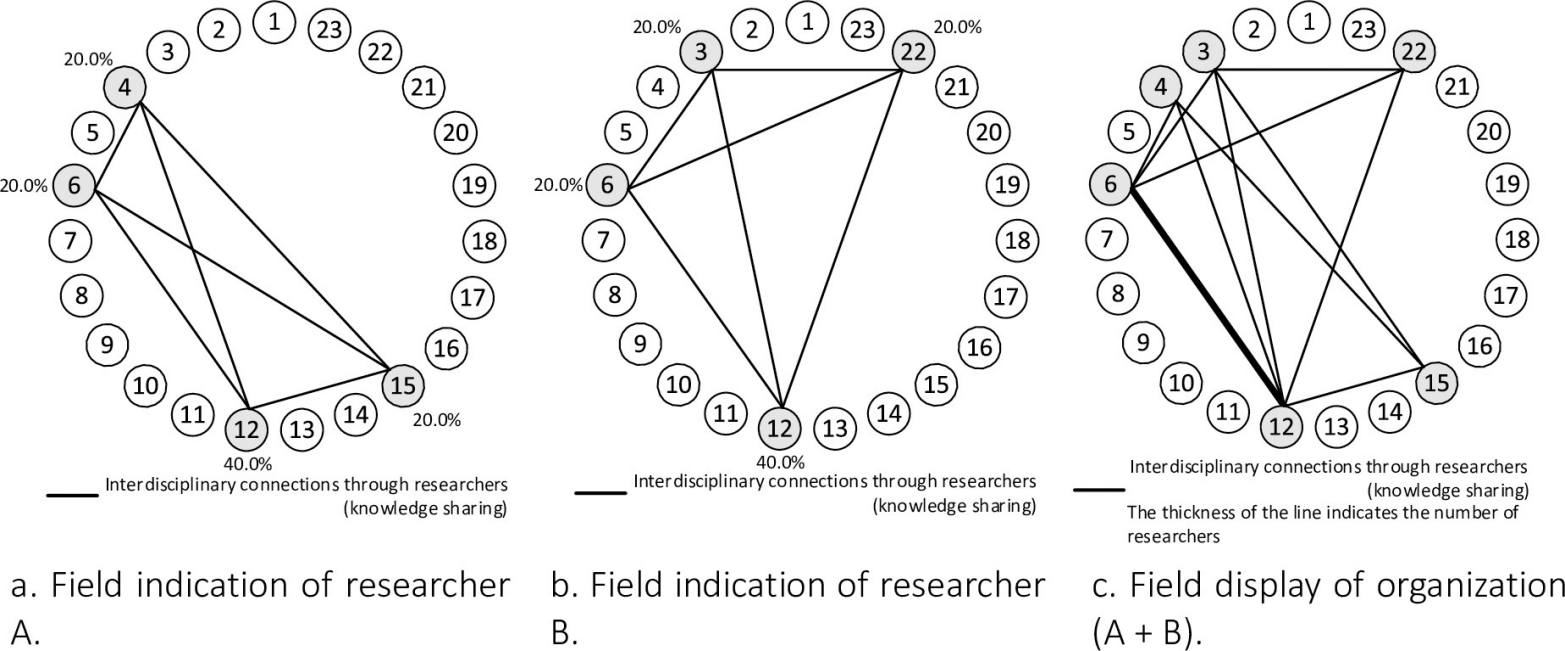
Researchers’ and an organization’s fields of view.

The information on each researcher’s field of expertise shows the method for visualizing an organization’s research capability and degree of cross-disciplinary integration; Fig 2 shows its application and overlays the information on researchers within the organization. However, the information for each researcher in Fig 2 does not show the connection between research fields without passing each researcher at the center, and it is difficult to recognize this connection. Therefore, we first used a simplified method for displaying the researcher’s field, where each researcher’s information is recombined into only the information between fields. Fig 3A shows the simplified field display method for researcher A. For example, clinical medicine (4) and mathematics (12) are connected through researcher A, and knowledge is shared. Thus, Fig 3A shows how each field is linked through researcher A.

Next, Fig 3C superimposes researcher A in Fig 3A and researcher B in Fig 3B to show the organization’s research capabilities and cross-disciplinary integration. The thick line between business (6) and mathematics (12) in Fig 3C superimposes the respective connections of Researcher A and Researcher B, while the thin line between the other fields are links through either researcher A or researcher B. Thus, the connections between the disciplines via researcher A in Fig 3A and researcher B in Fig 3B are clearly shown. In the method for visualizing organizational research power and interdisciplinary integration, the connecting lines are thicker because of the understanding that knowledge sharing between disciplines is enhanced based on the number or ratio of mediating researchers. This chart of the organization’s research capabilities and cross-disciplinary integration can be used for comparison between organizations.

In cross-organizational collaborations such as international coauthored papers, authors have different affiliations. In this case, this paper considers each author as contributing to the organization to which they belong, such as country or region.

### Classification based on similarities in the interdisciplinary connections of organizations

Hierarchical cluster analysis method is commonly used for finding subgroups. This method creates a dendrogram based on the similarity of the items analyzed. The researcher can choose where to cut the dendrogram to create clusters. This method does not have a generally accepted stopping rule for researchers to find the best set of clusters [25]). Procedures for hierarchical cluster analysis include single, complete, average linkages, as well as Ward’s method. Of these, Ward’s method is the most frequently used for bibliometric analysis; McCain [31] stated that both complete linkage and Ward’s method produce similar interpretable results.

Research papers with high similarity in interdisciplinary connections of organizations are gathered and grouped. In the classification process, we conducted a hierarchical cluster analysis using interdisciplinary connections as a variable for each organization and visualized the results using a dendrogram. Ward’s method was used to determine the distance between clusters (Fig 8). The interdisciplinary connections that characterize each group were color-coded by groups and visualized in a single graph (Fig 9). Next, principal component analysis was conducted for each group to show the patterns of interdisciplinary integration that constitute the group (Table 3). Then, the interdisciplinary connections characteristic of each pattern were color-coded for each group and visualized in a single graph (Fig 10).

**Table 3:** Contribution of the connections between artificial intelligence fields in the principal component analysis (2018).

Gr.	Principal Components	1	2	3	4	5	6	7	8	9	10	Analysis Objects*^1^
1	CR	1.000	0.000	0.000	0.000	0.000	0.000	0.000	0.000	0.000	0.000	1
	CCR	1.000	1.000	1.000	1.000	1.000	1.000	1.000	1.000	1.000	1.000	
2	CR	0.768	0.232	0.000	0.000	0.000	0.000	0.000	0.000	0.000	0.000	1
	CCR	0.768	1.000	1.000	1.000	1.000	1.000	1.000	1.000	1.000	1.000	
3	CR	0.398	0.248	0.169	0.135	0.050	0.000	0.000	0.000	0.000	0.000	3
	CCR	0.398	0.646	0.815	0.950	1.000	1.000	1.000	1.000	1.000	1.000	
4	CR	0.340	0.268	0.148	0.090	0.070	0.040	0.026	0.018	0.000	0.000	3
	CCR	0.340	0.680	0.756	0.846	0.916	0.956	0.982	1.000	1.000	1.000	

*Note*. CR: contribution rate, CCR: cumulative contribution rate. *^1^ Up to principal components with a CR of at least 0.10 and a CCR of at least 0.700.

This process is considered effective in simplifying the characteristics of each group when interpreting the results.

## Analysis

### Analysis procedure

**Fig 4:**
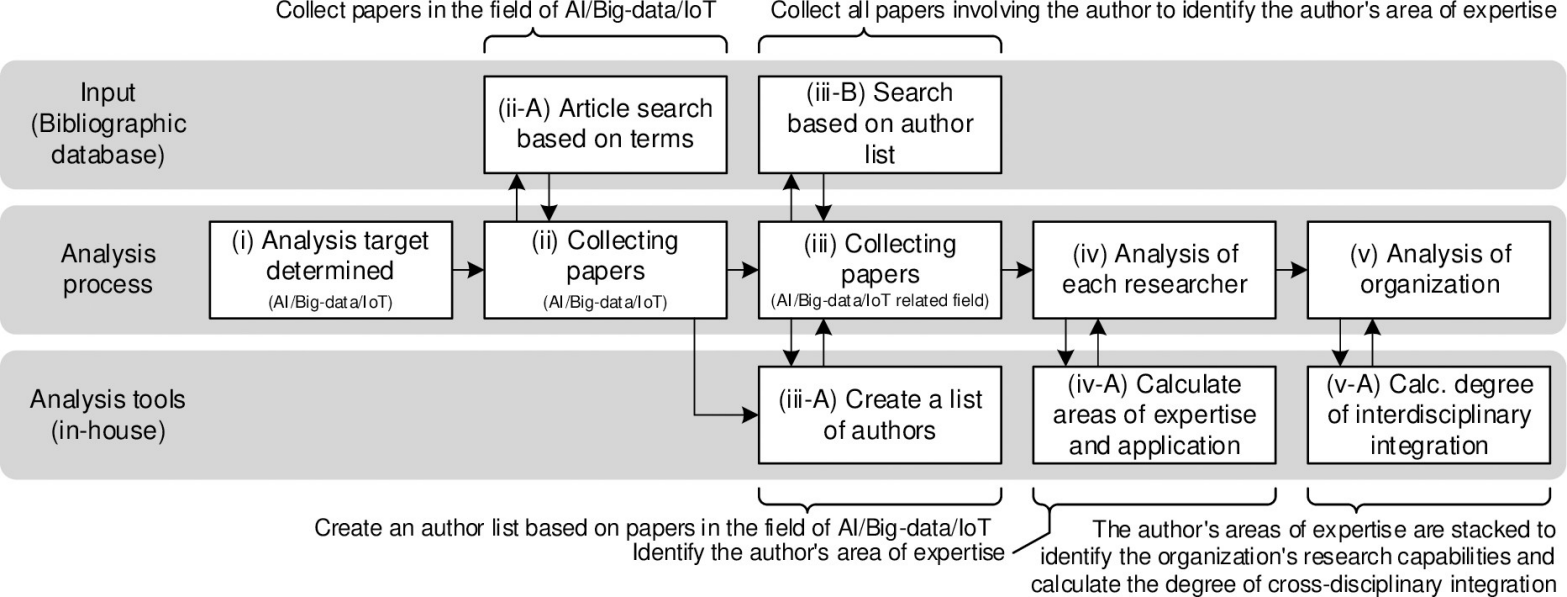
Procedure for analyzing an organization’s research capabilities and interdisciplinary integration. *Note*: Only the procedure of the first step is shown. The second step is to perform the analysis with the statistical analysis software "R".

The proposed analysis has three stages (Fig 4): “analysis of the research capability of organizations and cross-disciplinary fusion,” “hierarchical cluster analysis by country focusing on the similarity of cross-disciplinary fusion,” and “principal component analysis to quantitatively understand the factors of the classification of cross-disciplinary fusion.”

First, we determined the field to be analyzed (i.e., field A (i) in Fig 4). Next, we collected papers in field A (ii and ii-A) and gathered those in which the authors were involved to identify their fields of expertise in field A (iii). We then extracted the names all authors from the papers in the previous step (ii) (iii-A) and collected all papers written by these authors in the relevant year (iii-B). Subsequently, we determined all authors’ fields of expertise (iv and iv-A). Finally, we identified the organizations’ research capabilities and calculated their degree of interdisciplinary integration (v and v-A).

Second, we conducted hierarchical cluster analysis on 253 types of interdisciplinary connections and visualized it through a dendrogram. Third, principal component analysis (PCA) was conducted on the same interdisciplinary connections to extract classification factors and quantify country-specific characteristics. The data were collected from the WoS Core Collection, and author extraction was performed using in-house software based on Microsoft Visual Basic for Applications. Both PCA and clustering analysis were conducted using statistical analysis software R.

### Collected data

**Fig 5:**
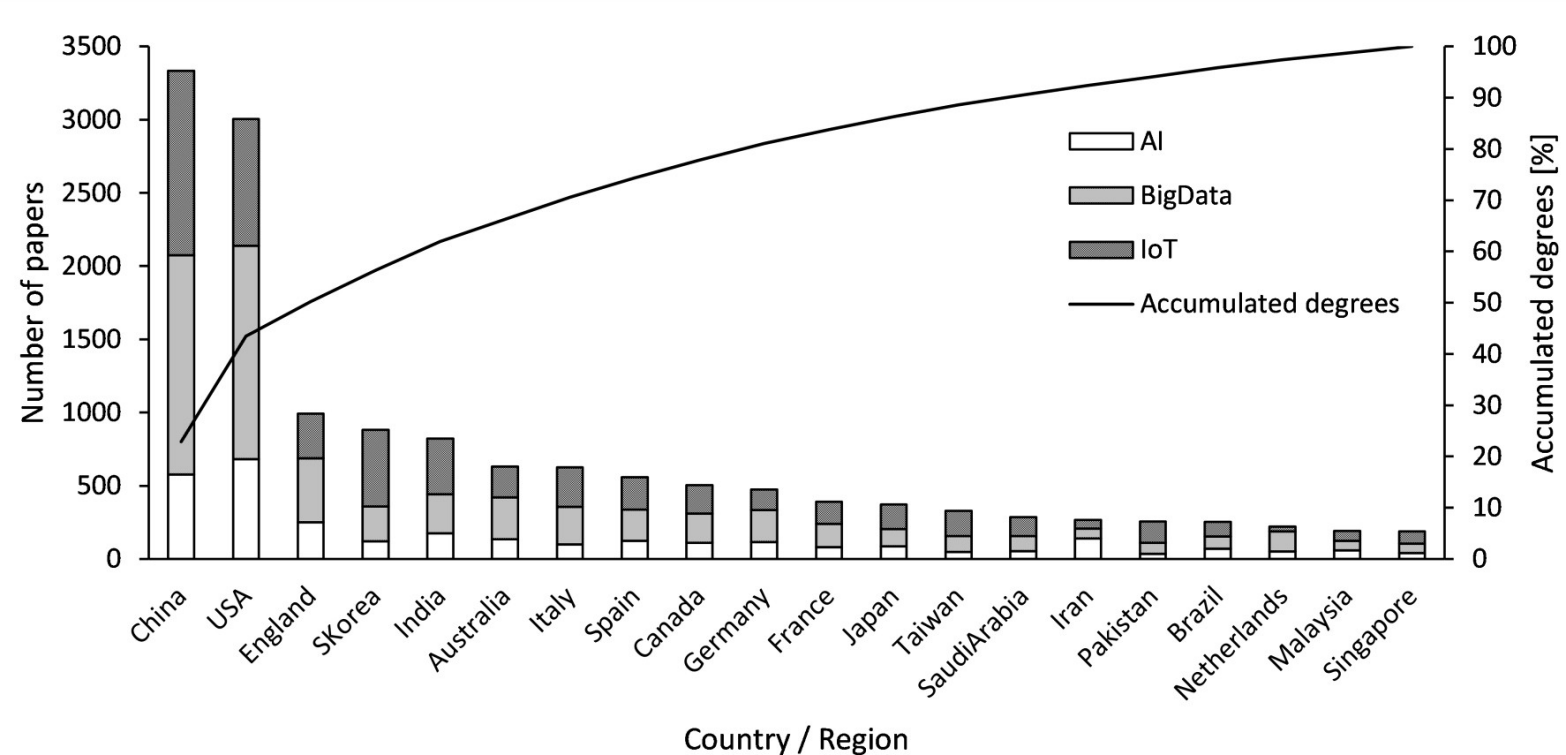
Top 20 countries by number of papers (2018). *Note*. Country rankings are by the total number of papers in AI, big data, and IoT.

The number of papers on AI, big data, and IoT continuously increased to the values of 2,843, 5,118, and 4,661, respectively, by 2018. Fig 5 presents a comparison of the papers in top 20 countries in 2018. The country rankings were based on the total number of studies on the three technologies. Additionally, the number of papers for each country was counted according to the countries of all authors, and not just the responsible author, and more than one country in the case of international co-authorship. This method was adopted because “responsible author” carries different definitions depending on the research field, and in some cases, more than one responsible author is registered in the WoS. However, if there are multiple co-authors in the same country, the same paper will be counted as one paper to avoid counting the number of co-authors.

### Analytical methods and results

#### Collection of papers and extraction of authors in AI/big data/IoT

In steps (i) and (ii), articles on AI, big data, and IoT were collected. The search criteria included the document type (“article” or “review”), year of publication (2018), language (English), and different topics (“AI” or “artificial intelligence,” “big data” or “bigdata,” and “IoT” or “Internet of Things”).

In step (iii), we determined the author count based on the papers in each field and found 13,203 for AI, 26,977 for big data, and 19,836 for IoT in top 20 countries in 2018. The United States ranked first with 12,744 authors (21.2%), followed by China (12,662 authors, 21.1%) and England (4,397 authors, 7.3%). The United States and China were the only countries exceeding 10.0% of the total number of authors, accounting for 42.3%.

In the person data in this study, the same name and surname shall be considered as the same person. If they are from different countries, they shall be considered different persons. As the analysis in this study is characterized by trends by country, it is assumed that some confusion of the same name occurs in each country/region. This is a limitation of this study.

### Collection of papers in related fields and identification of authors’ areas of expertise

**Fig 6:**
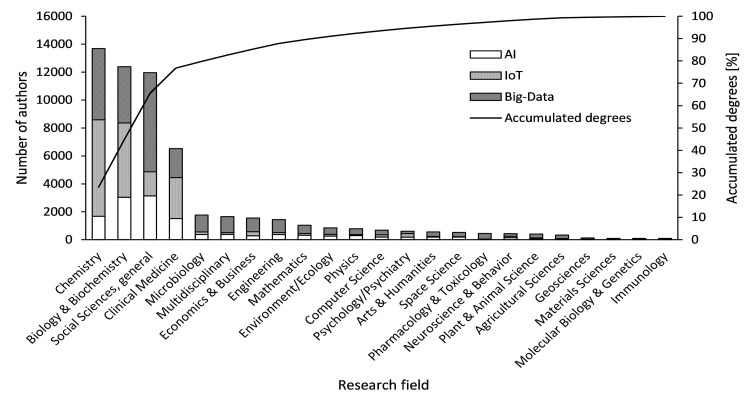
Specialties of authors from the top 20 countries (2018).

Step (iv) identifies the authors’ areas of expertise following the method by Mizukami et al. [7]. Based on the list of authors in step (iii), we collected all related papers published by all authors in the past three years (2016, 2017, and 2018). From the extracted papers, we then identified the researchers’ areas of expertise. Fig 6 shows the distribution of fields of expertise, which were identified for 58,037 authors.

For the field of study, chemistry ranked first with 13,683 authors (23.7%), followed by biology and biochemistry (12,389 authors, 21.3%) and general social sciences (11,954 authors, 20.6%), accounting for 65.5% of the total. The only other field above 10.0% was clinical medicine.

#### Extracting links between research areas and similarities by country

**Fig 7:**
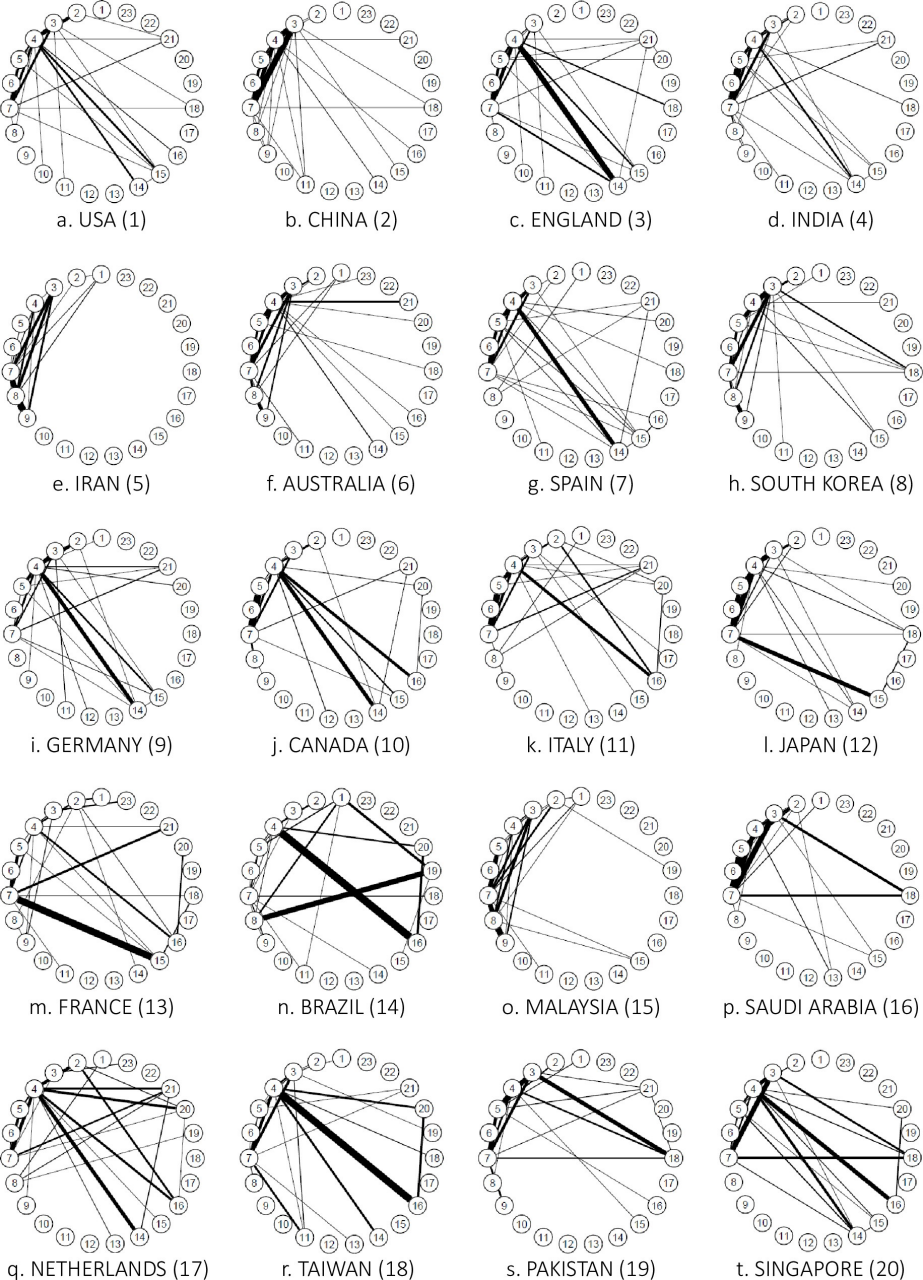
Connections between author research fields in the Top 20 countries for artificial intelligence (2018). *Note*: This indicates connections with a strength of 1.0% or more.

*AI*. Fig 7 shows the connections between the research fields of the top 20 countries in terms of the number of AI studies and a linkage strength of 1.0% or more.

In AI, the United States, China, and England were the top three countries, in that order. The United States has a complete network of chemistry, clinical medicine, and engineering (3–4–7) and clinical medicine, computer science, and engineering (4–5–7). Links were also observed between biology and biochemistry and chemistry (2–3), between clinical medicine and molecular biology and genetics (4–14), and between clinical medicine and multidisciplinary (4–15). Because the United States does not have any significantly strong links, numerous broad links can be assumed. China has complete networks in chemistry, clinical medicine, and engineering (3–4–7) and clinical medicine, computer science, and engineering (4–5–7), while England has complete networks in clinical medicine, computer science, and engineering (4–5–7) and clinical medicine, engineering, and molecular biology and genetics (4–7–14). Connections were also observed between chemistry and engineering (3–7), clinical medicine and multidisciplinary (4–15), and clinical medicine and physics (4–18).

**Fig 8:**
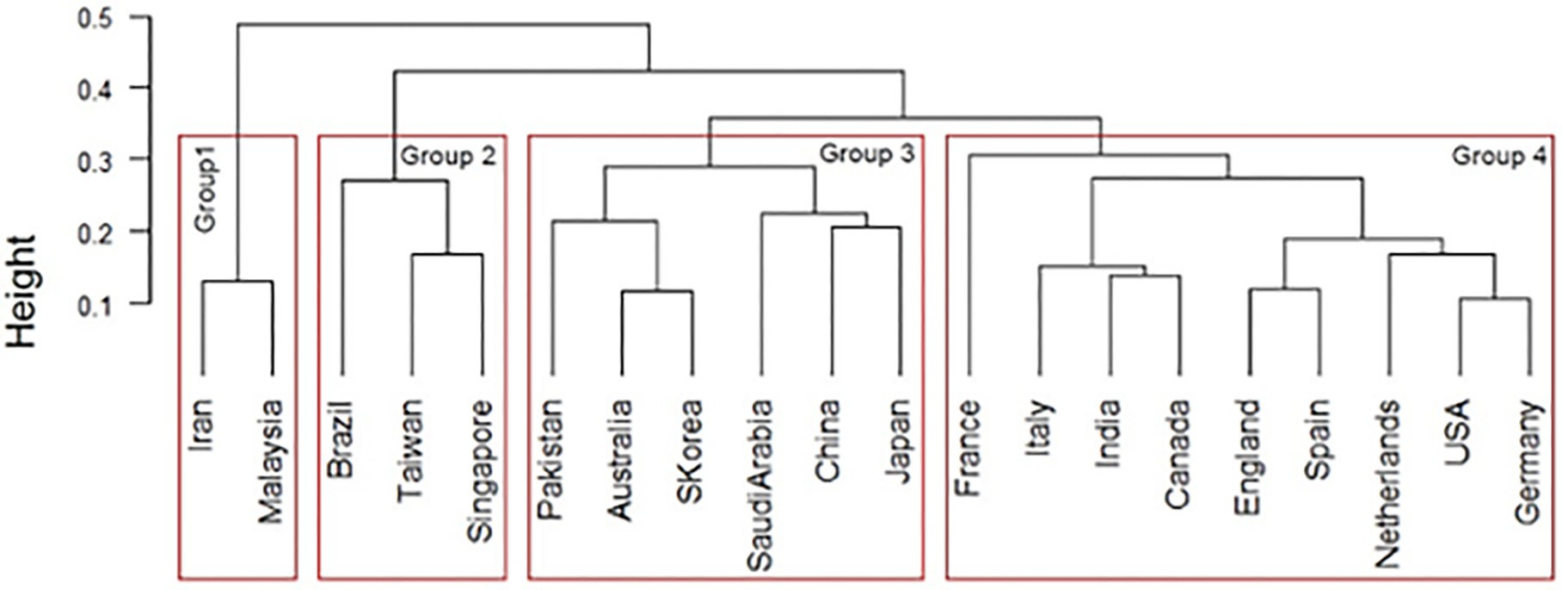
Similarity dendrograms by country: Links between author research fields in the top 20 countries for artificial intelligence. *Note*. Distance: hclust (*, “ward.D2”).

We then performed hierarchical cluster analysis on the interdisciplinary connections of the top 20 countries. We used country as an individual and interdisciplinary connections as a variable, and visualized them using a dendrogram (Fig 8). Ward’s method was followed to determine the distance between clusters.

As shown in Fig 8, the top 20 AI countries were classified into four groups for the ease of interpretation: Group 1 consists of Islamic countries such as Iran and Malaysia; Group 2 consists of South American and Southeast Asian countries with developed economies such as Brazil, Taiwan, and Singapore; Group 3 consists of Asian Oceania countries such as Pakistan, Australia, South Korea, Saudi Arabia, China, and Japan; and Group 4 consists of France, Italy, India, Canada, England, Spain, the Netherlands, the United States, and Germany. Notably, Group 3 includes Middle Eastern countries with strong economic ties with countries within the Pacific Rim, while Group 4 constitutes South Asian countries with historically strong relations with the English-speaking world. In addition, each group has subgroups that are particularly similar. For example, when the level is set to 0.25, groups 2, 3, and 4 have subgroups.

**Fig 9:**
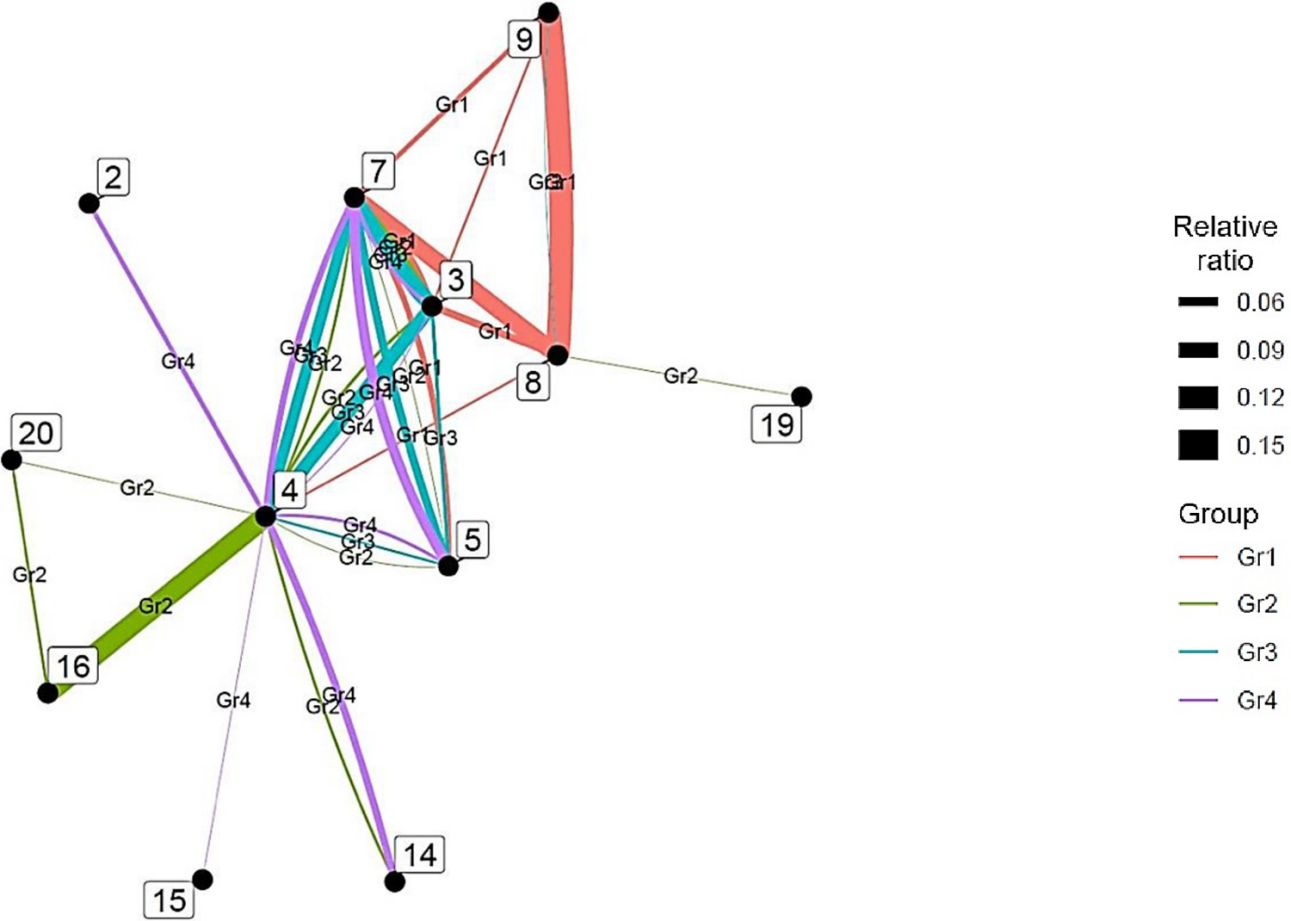
Distinctive interdisciplinary connections for each artificial intelligence group.

Next, we clarified the characteristic interdisciplinary connections for each group (Fig 9), excluding those with less than 0.030 of the total number of authors. This method simplifies these relations when interpreting the results.

In Group 1, interdisciplinary connections are strong between engineering and environment/ecology (7–8) and environment/ecology and geosciences (8–9). In addition, chemistry, engineering, and environment/ecology (3–7–8) have a complete network that links all research fields. In Group 2, clinical medicine and neuroscience and behavior (4–16) have strong interdisciplinary connections. Further, Group 3 is characterized by strong interdisciplinary connections among clinical medicine (4), chemistry (3), computer science (5), engineering (7), molecular biology and genetics (14), and psychology/psychiatry (20), with the first four constituting a complete network connecting all research fields (3–4–5–7). Finally, in Group 4, clinical medicine, computer science, and engineering (4–5–7) are a complete network connecting all research fields, and clinical medicine (4), biology and biochemistry (2), molecular biology and genetics (14), and multidisciplinary (15) are interconnected.

Furthermore, PCA was conducted on each group’s interdisciplinary connections to clarify their composition. A variance–covariance matrix was used, considering country as an individual and interdisciplinary linkage as a variable of the interdisciplinary linkages of each group to extract the classification factors and show their characteristics. The number of principal components (M) to be analyzed for each group was defined as having a contribution rate (CR) of 0.10 or higher and whose cumulative contribution rate (CCR) was 0.700 or higher (Table 3).

In Group 1, the CR was 1.000 from the first principal component and 0.000 below. Only the first principal component was analyzed. Next, for each principal component in Group 2, the CRs were 0.768, 0.232, and 0.000 from the first principal component. Only the first principal component was examined as well, and the CCR was 0.768. In Group 3, the CRs were 0.398, 0.248, 0.169, 0.135, 0.050, and 0.000 from the first principal component. Here, the third principal component was analyzed, and the CCR was 0.815. Finally, in Group 4, the CRs were 0.340, 0.268, 0.148, 0.090, 0.070, 0.040, 0.026, 0.018, and 0.000 from the first principal component. Only the third principal component was analyzed as well, and the CCR was 0.756.

In Groups 1 and 2, only principal component 1 was analyzed; in groups 3 and 4, only principal component 3 was examined. For groups 3 and 4, the characteristics of each principal component are presented to show the tendency of interdisciplinary connections within the groups. The number of principal components to be analyzed (M) has been determined; the next step is to exclude the variables unrelated to the principal components. This is considered effective for simplifying the relations when interpreting the results. We assume that the variables unrelated to 1 to M principal components are unnecessary. Given the variable ${\overset{ˇ}{X}}_{k}$, its correlation coefficient with the principal component ${\overset{ˇ}{t}}_{j}$ is expressed in Eq 1:

Correlation coefficient of principal component ${\overset{ˇ}{t}}_{j}$ of variable ${\overset{ˇ}{X}}_{k}$:

$$r_{kj}=\sqrt{\lambda_{j}}a_{kj}/\sqrt{var[{\overset{ˇ}{X}}_{k}]}$$ (1)

Eq 2 shows the cumulative coefficient of determination:

Cumulative coefficient of determination up to the principal component ${\overset{ˇ}{t}}_{M}$ of variable ${\overset{ˇ}{X}}_{k}$:

$$\sum_{j=1}^{M}r_{kj}^{2}=\frac{1}{var[{\overset{ˇ}{X}}_{k}]}\sum_{j=1}^{M}\lambda_{j}a_{kj}^{2}$$ (2)

Therefore, variable with smaller values of coefficients may be excluded. Note that $var[{\overset{ˇ}{X}}_{k}]$ = 1 if we consider the data in terms of the correlation matrix using standardized variables and not the variance–covariance matrix. Here, variables with a cumulative coefficient of determination of less than 0.300 up to the principal component M were excluded.

**Fig 10:**
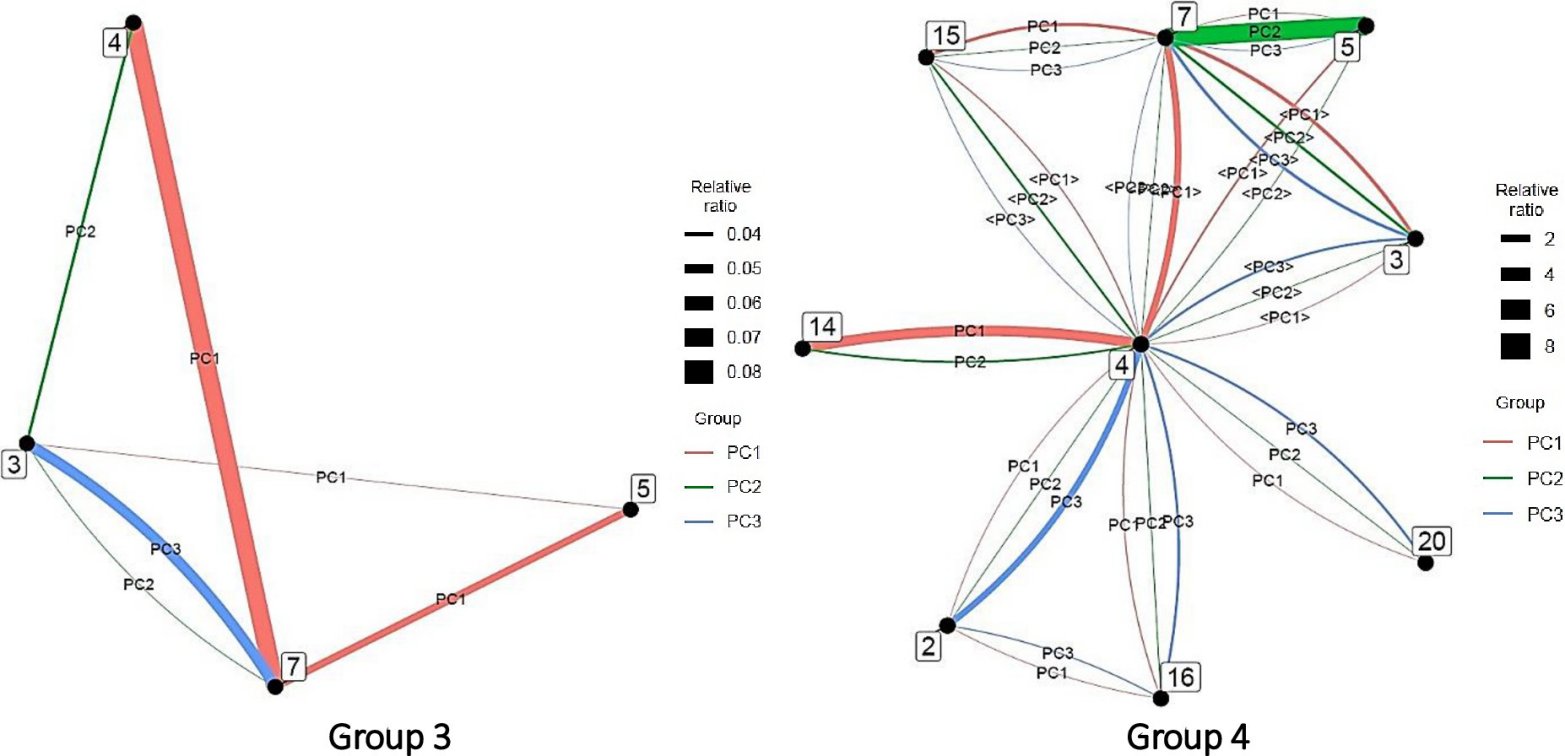
Principal component analysis for each artificial intelligence group. *Note*. <PC#> has factor loadings below 0.300 but are shown as reference information.

In this study, a vector of factor loadings of 0.700 or more was considered a strong influence of the factor on the variable, 0.300 or more as moderate, and below 0.300 as weak. Variables for which the vector of factor loadings was below 0.300 were excluded. Fig 10 shows the PCA results for AI.

In Group 3, the first principal component was centered on computer science (5) and engineering (7), with clinical medicine and engineering (4–7), computer science and engineering (5–7), and computer science and chemistry (5–3); the second main component focused on chemistry (3) and was characterized by interdisciplinary links with clinical medicine (3–4) and engineering (3–7); and the third main component was depicted by the interdisciplinary links between chemistry and engineering (3–7).

In Group 4, the first main component of was indicated by multiple interdisciplinary links with clinical medicine (4) at the center and its connection with molecular biology and genetics (4–14) at the head. The second main component was similar to that of its first main component but was characterized by the concentration of authors in computer science and engineering (5–7). The third main component was also similar to that of its first main component but was indicated by the concentration of authors in clinical medicine and biology and biochemistry (4–2).

**Fig 11:**
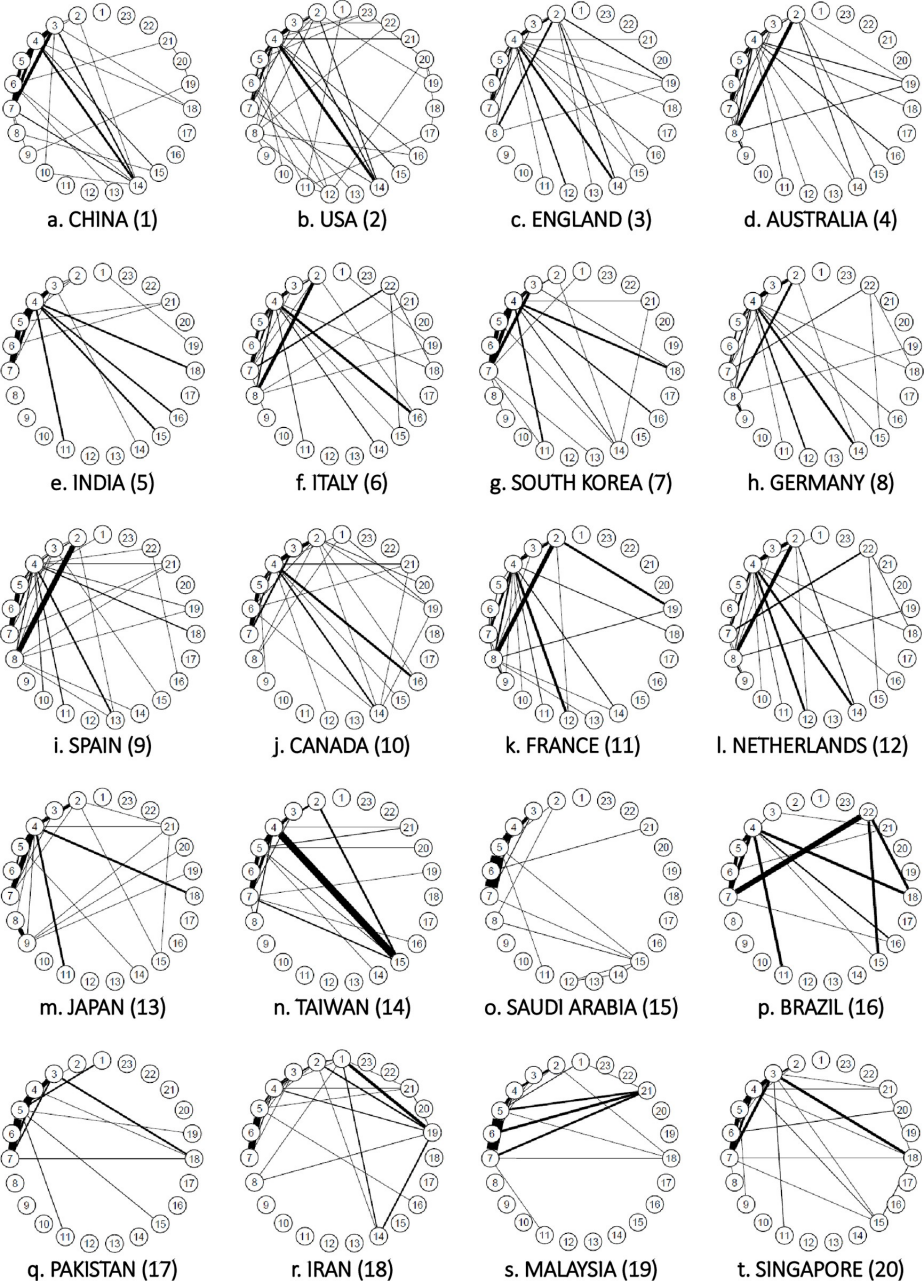
Connections between author research fields in the top 20 big data countries (2018). *Note*. This indicates connections with a strength of 1.0% or more.

*Big data*. Fig 11 shows the connections between the research fields of the top 20 countries in terms of the number of big data papers, the top three being China, the United States, and England. China ranked first, with a complete network of chemistry, clinical medicine, and engineering (3–4–7); clinical medicine, computer science, and engineering (4–5–7); and chemistry, clinical medicine, and molecular biology and genetics (3–4–14). The United States ranked second, with clinical medicine, computer science, and engineering (4–5–7); chemistry, clinical medicine, and molecular biology and genetics (3–4–14); and biology, biochemistry, and chemistry (3–4–14). Biology and biochemistry, chemistry, and molecular biology and genetics (2–3–14) were complete networks. Clinical medicine and general social sciences (4–21) were also connected. The United States has a wide range of connections but none was strong. England, in the third place, has numerous connections, mainly in clinical medicine (4) and biology and biochemistry (2) and is considered to have many and wide connections with no exceptionally strong ones.

**Fig 12:**
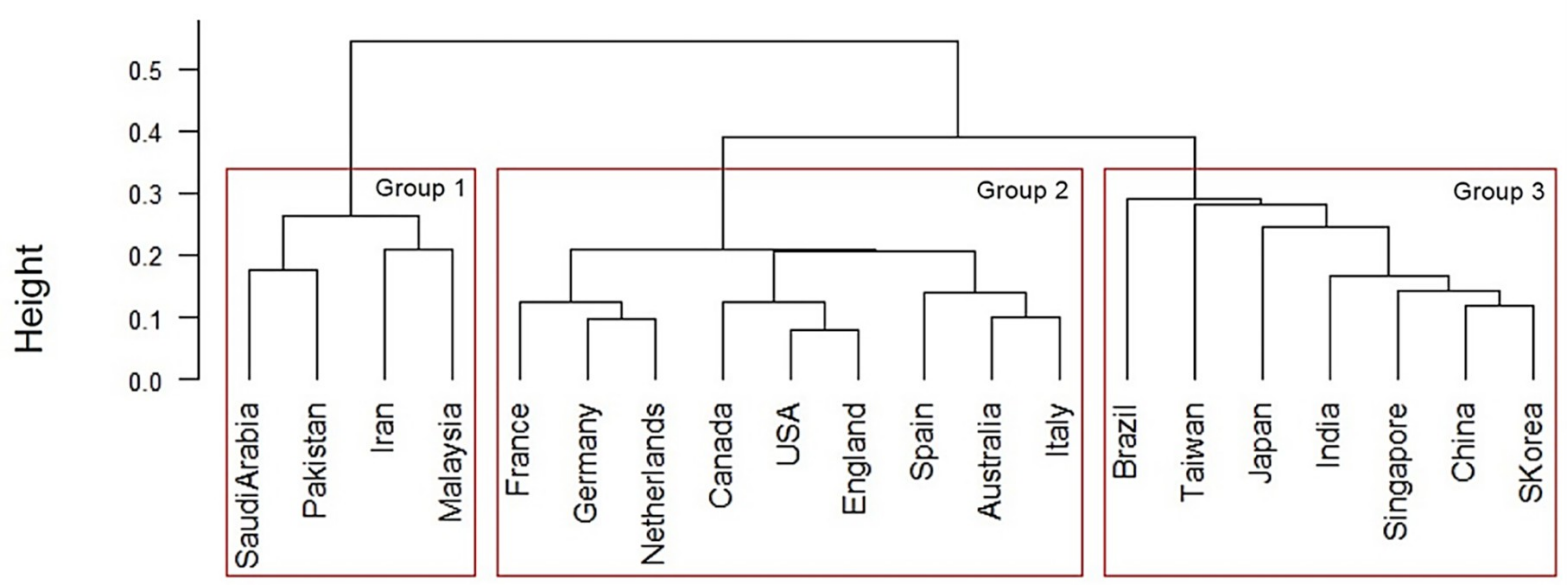
Similarity dendrogram by country: Links between author research areas in the top 20 big data countries. *Note*. Distance: hclust (*, “ward.D2”).

The interdisciplinary links of the top 20 countries were analyzed using hierarchical cluster, using countries as individuals and interdisciplinary links as variables and visualized using a dendrogram (Fig 12). The cluster analysis for big data was the same as that for AI.

In Fig 12, the top 20 big data countries were classified into three groups for the ease of interpretation: Group 1 consisted of Saudi Arabia, Pakistan, Iran, and Malaysia. Group 2 included France, Germany, the Netherlands, Canada, the United States, England, Spain, Australia, and Italy. Group 3 comprised Brazil, Taiwan, Japan, India, Singapore, China, and South Korea.

**Fig 13:**
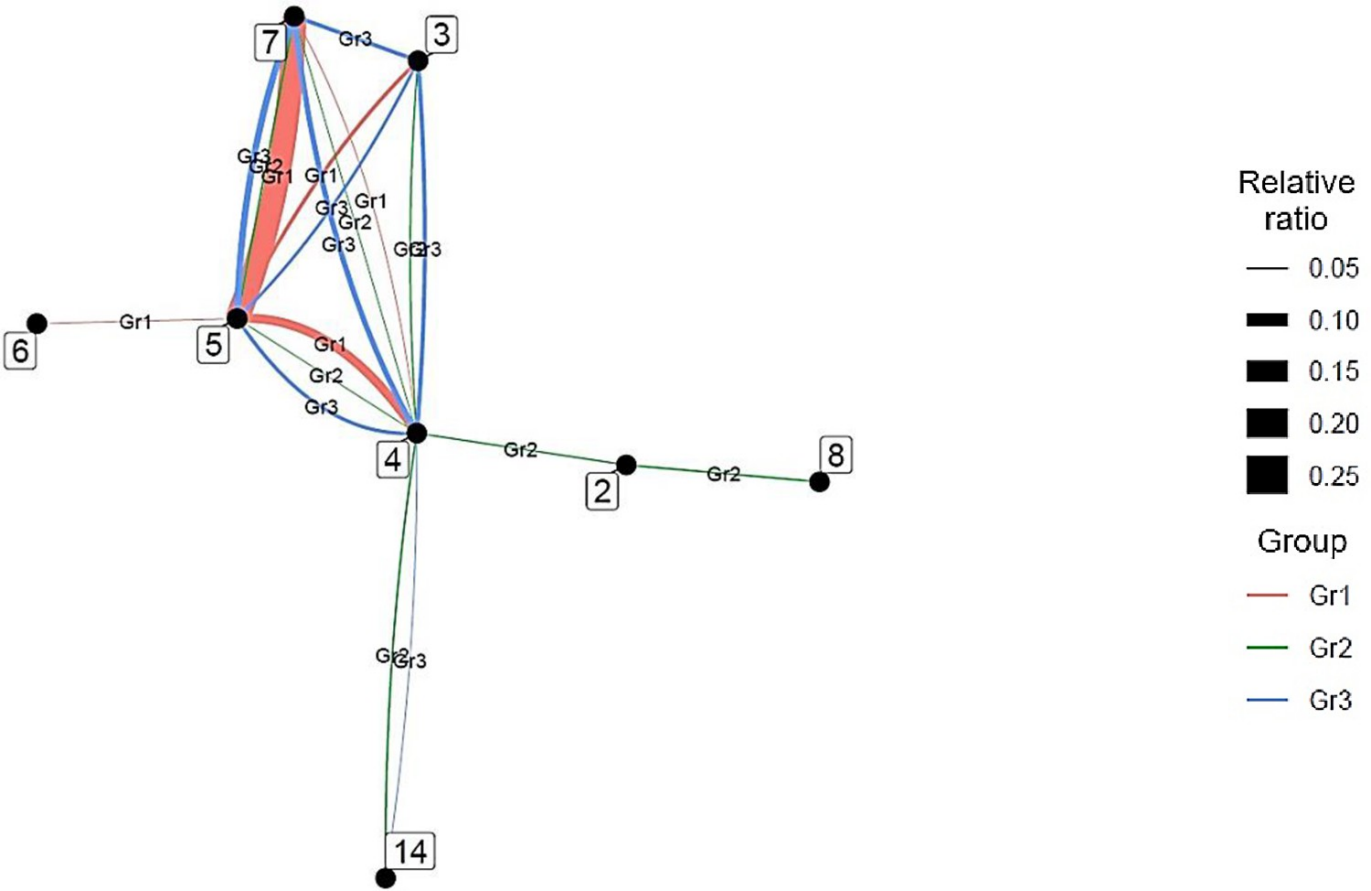
Distinctive interdisciplinary links of each big data group.

Furthermore, we clarified the characteristic interdisciplinary connections of each group in the dendrogram (Fig 13). The method for displaying interdisciplinary connections in big data was the same as the threshold used in the AI analysis.

In Group 1, the focus was on computer science (5), followed by computer science and engineering (5–7), clinical medicine and computer science (4–5), and chemistry and computer science (3–5). Group 2 had several connections, especially in clinical medicine (4). In addition, as there were no exceptionally strong connections, we can assume numerous broad connections. Finally, in Group 3, chemistry, clinical medicine, computer science, and engineering (3–4–5–7) were found to have a complete network connecting all research fields.

Next, we performed PCA on each group’s interdisciplinary connections to clarify their composition using the variance–covariance matrix, with country as an individual and interdisciplinary connection as a variable. The selection of the number of principal components (M) for each group was the same as that in the AI analysis. Table 4 shows the CR of each principal component in each group’s PCA.

**Table 4:** Contribution of big data to the principal component analysis of connections between research fields (2018).

Gr.	Principal Components	1	2	3	4	5	6	7	8	9	10	Analysis Objects*^1^
1	CR	0.524	0.349	0.127	0.000	0.000	0.000	0.000	0.000	0.000	0.000	2
	CCR	0.524	0.873	1.000	1.000	1.000	1.000	1.000	1.000	1.000	1.000	
2	CR	0.344	0.328	0.105	0.080	0.061	0.051	0.021	0.012	0.000	0.000	3
	CCR	0.344	0.672	0.776	0.857	0.917	0.968	0.988	1.000	1.000	1.000	
3	CR	0.346	0.252	0.211	0.093	0.067	0.031	0.000	0.000	0.000	0.000	3
	CCR	0.346	0.598	0.809	0.902	0.969	1.000	1.000	1.000	1.000	1.000	

*Note*. CR: contribution rate, CCR: cumulative contribution rate. *^1^ Up to principal components with a CR of at least 0.10 and a CCR of at least 0.700.

In Group 1, the CRs were 0.524, 0.349, 0.127, and 0.000 from the first principal component. The second principal component was also included in the analysis, and the CCR was 0.873. Subsequently, in Group 2, the CRs were 0.344, 0.328, 0.105, 0.080, 0.061, 0.051, 0.021, 0.012, and 0.000 from the first principal component. The third principal component was analyzed; the CCR was 0.776. Finally, in Group 3, the CRs were 0.346, 0.252, 0.211, 0.093, 0.067, 0.031, and 0.000 from the first principal component. The third principal component was examined; the CCR was 0.806.

**Fig 14:**
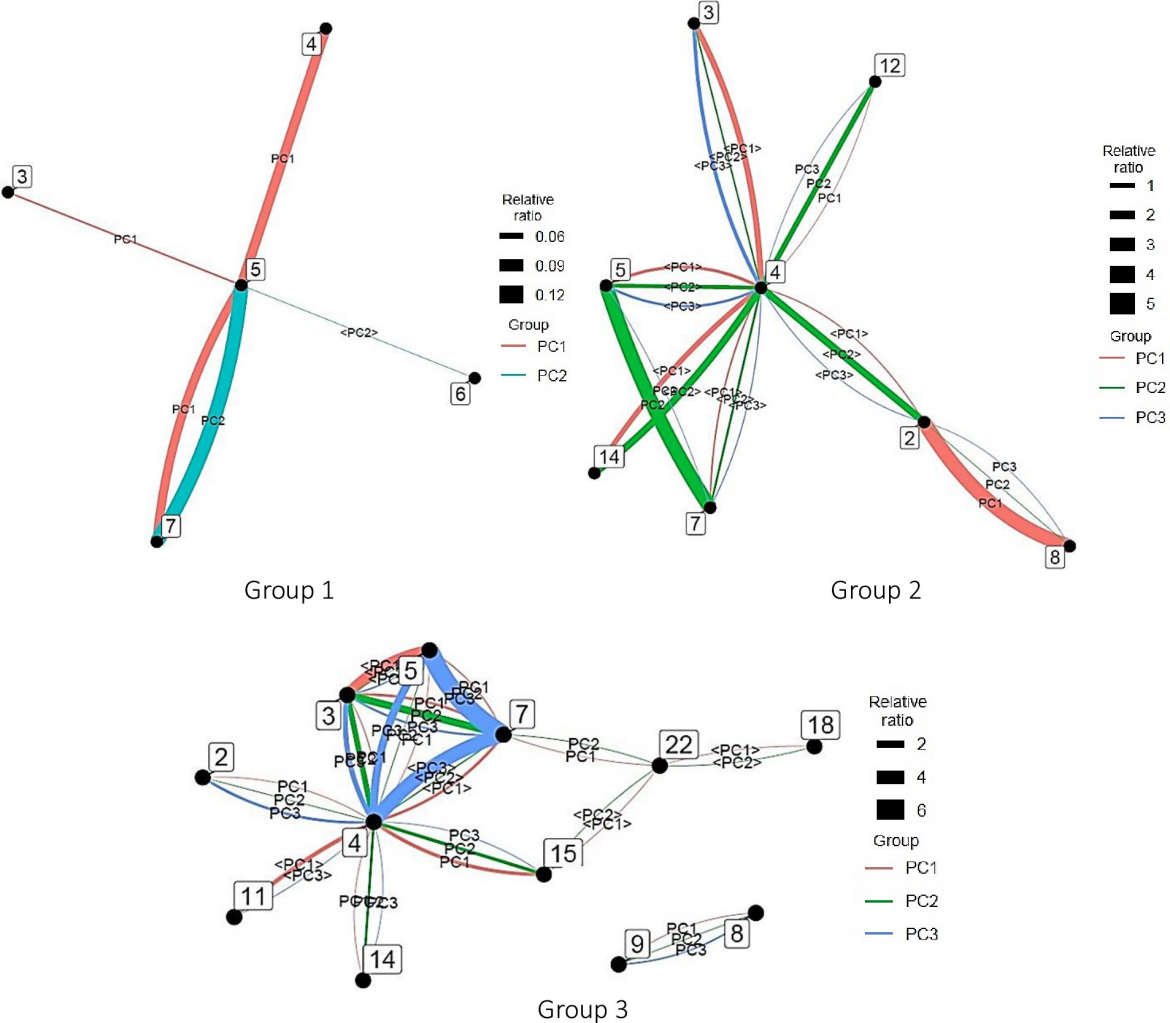
Principal component analysis of each group in big data. *Note*. <PC#> has factor loadings below 0.300 but are shown as reference information.

The characteristics of each principal component were presented to show the tendency of the connection between fields within each group. The analysis method is similar to that of AI analysis. Fig 14 shows the PCA results for big data.

In Group 1, the first principal component was computer science (5), followed by computer science and chemistry (5–3), computer science and clinical medicine (5–4), and computer science and engineering (5–7), while the second was computer science (5) and depicted by its interdisciplinary links with engineering (5–7) and economics and business (5–6).

In Group 2, the first major component was indicated by interdisciplinary links between biology and biochemistry and environment/ecology (2–8). Its second and third main components were characterized by the interdisciplinary links between computer science and engineering (5–7) and clinical medicine and mathematics (4–12) and between computer science and engineering (5–7), clinical medicine and mathematics (4–12), and biology and biochemistry and environment/ecology (2–8), respectively. However, as there were no author-intensive links, numerous broad connections likely exist.

In Group 3, the first principal component was centered on engineering (7) and characterized by its interdisciplinary connections with chemistry (7–3), computer science (7–5), and space science (7–22). The first main component of Group 3 centered on clinical medicine (4) and included clinical medicine and multidisciplinary (4–15), clinical medicine and biology and biochemistry (4–2), clinical medicine and molecular biology and genetics (4–14), and clinical medicine and computer science (4–5). The second main component focused on chemistry (3) and clinical medicine (4) and included chemistry and clinical medicine (3–4), chemistry and engineering (3–7), clinical medicine and multidisciplinary (4–15), and clinical medicine and molecular biology and genetics (4–14). The third main component consisted of computer science (5), clinical medicine (4), computer science and clinical medicine (5–4), computer science and engineering (5–7), clinical medicine and chemistry (4–3), and clinical medicine and biology and biochemistry (4–2).

**Fig 15:**
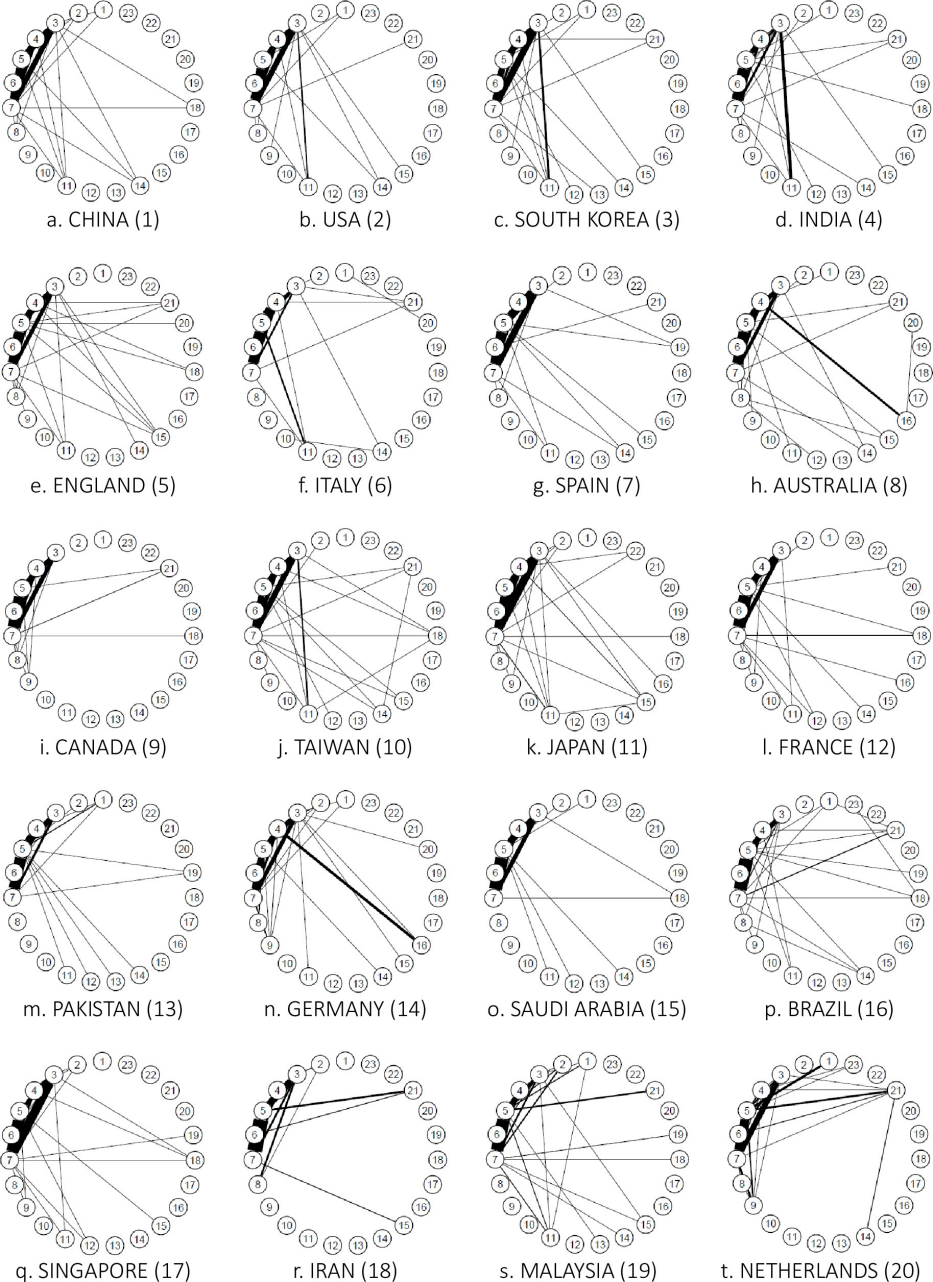
Connections between author research fields in the top 20 countries for internet of things (2018). *Note*. This indicates connections with a strength of 0.5% or more.

*IoT*. Fig 15 shows the links between the research fields of the top 20 countries in terms of the number of papers on IoT, indicating the strength of the links as 0.5% or more. In IoT, the top three countries were China, followed by the United States and South Korea.

China showed a complete network of chemistry, clinical medicine, and engineering (3–4–7) and clinical medicine, computer science, and engineering (4–5–7). Meanwhile, the United States displayed a complete network of chemistry, clinical medicine, and engineering (3–4–7) and clinical medicine, computer science, and engineering (4–5–7). The United States also showed interdisciplinary links between chemistry and materials sciences (3–11). South Korea also had a complete network of chemistry, clinical medicine, and engineering (3–4–7) and clinical medicine, computer science, and engineering (4–5–7). This was characterized by the interdisciplinary links between chemistry and materials sciences (3–11).

**Fig 16:**
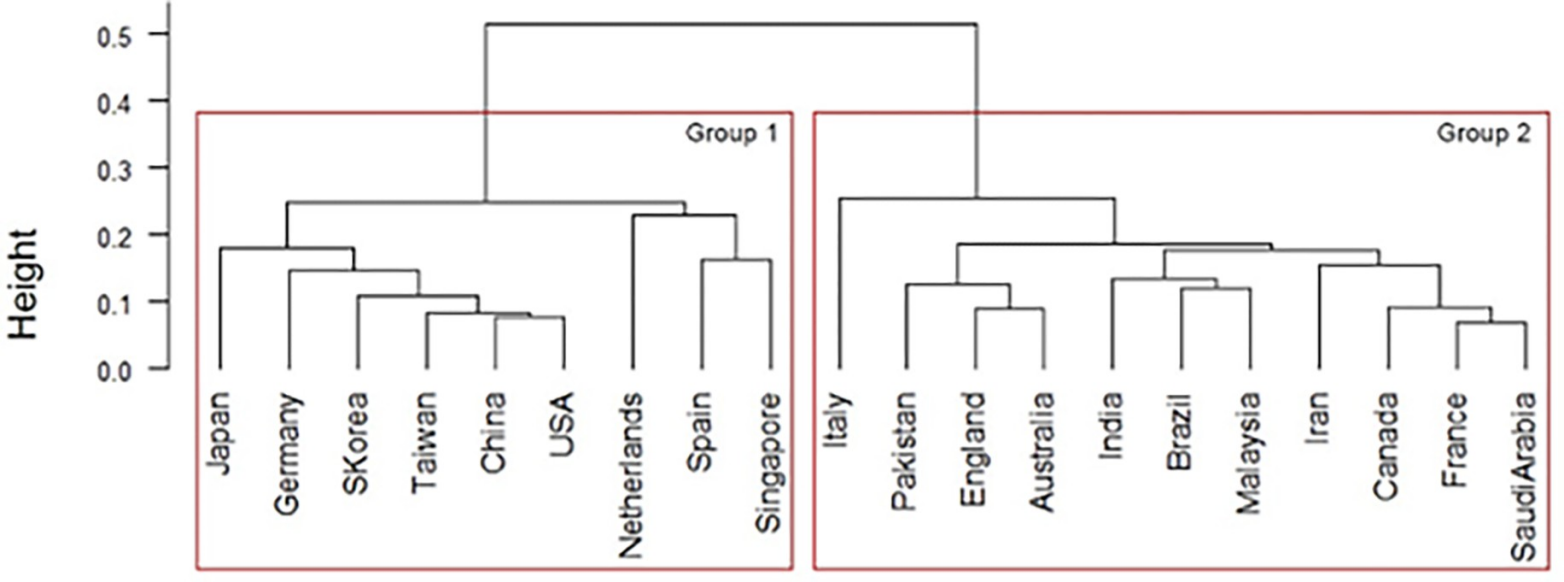
Similarity dendrogram by country: Links between author research areas in the top 20 countries for internet of things. *Note*. Distance: hclust (*, “ward.D2”).

Next, the hierarchical cluster analysis was applied to the interdisciplinary connections of the top 20 countries, using countries as individuals and interdisciplinary connections as variables and visualized in a dendrogram (Fig 16). The cluster analysis for IoT was the same as that for AI.

Fig 16 shows the top 20 IoT countries classified into two groups for the ease of interpretation: Group 1 consisted of Japan, Germany, South Korea, Taiwan, China, the United States, the Netherlands, Spain, and Singapore. Group 2 included Italy, Pakistan, England, Australia, India, Brazil, Malaysia, Iran, Canada, France, and Saudi Arabia.

**Fig 17:**
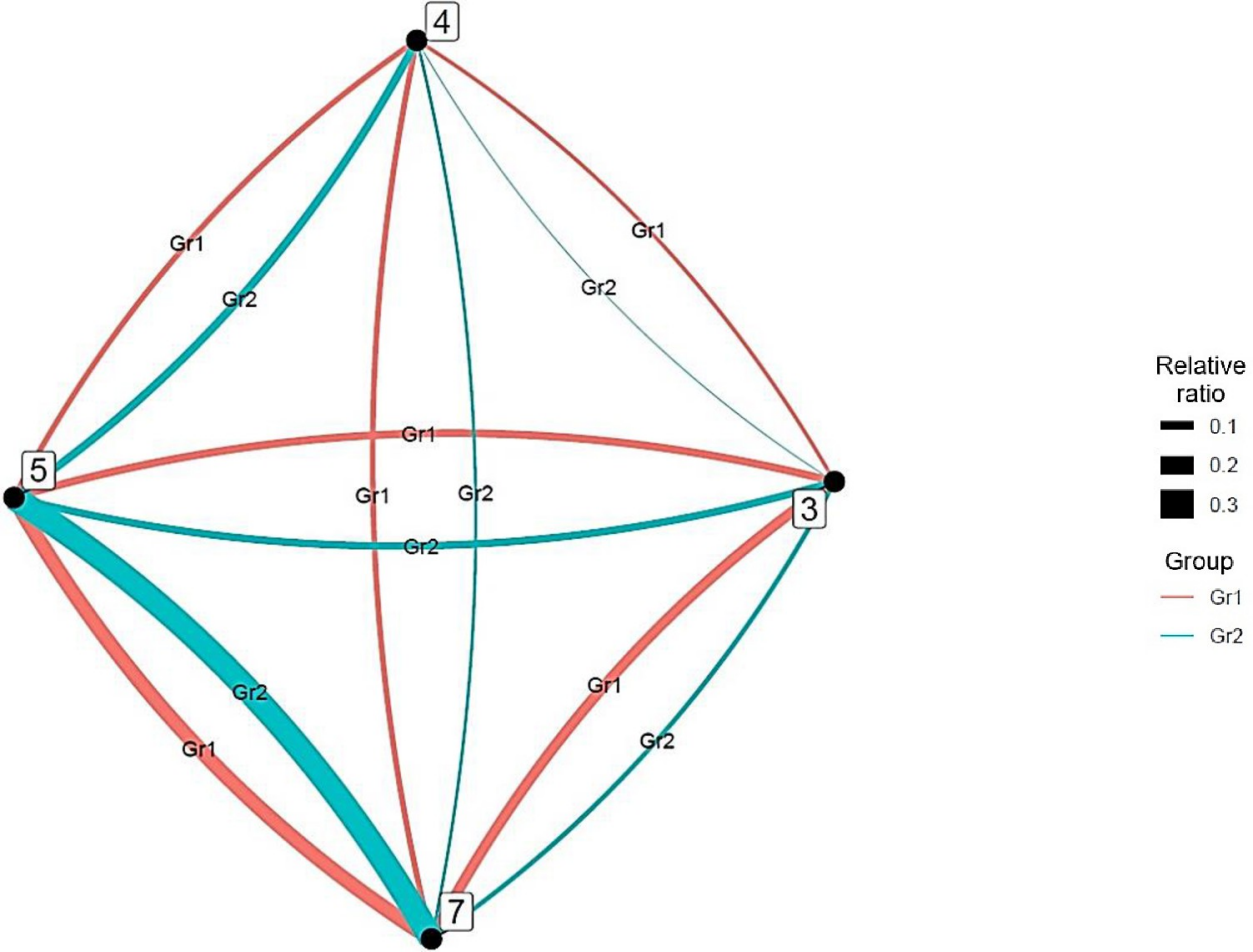
Distinctive interdisciplinary links of each internet of things group.

Furthermore, the dendrogram clarifies each group’s characteristic interdisciplinary connection (Fig 17). The method for displaying the interdisciplinary connections for IoT was the same as the threshold used in the AI analysis.

Groups 1 and 2 have a common characteristic, that is, chemistry, clinical medicine, computer science, and engineering (3–4–5–7) were complete networks connecting all research fields. Additionally, Group 1 was a complete network that linked all research fields and dominated by chemistry and engineering (3–7), while Group 2 tended to be dominated by computer science and engineering (5–7).

Next, PCA was applied to each group’s interdisciplinary connections to clarify their composition using the variance–covariance matrix, with country as an individual and interdisciplinary connection as a variable. The selection of the number of principal components (M) for each group was the same as that in the AI analysis. Table 5 shows the CR of each principal component in each group’s PCA.

**Table 5:** Contribution of internet of things to the principal component analysis of connections between research fields (2018).

Gr.	Principal Components	1	2	3	4	5	6	7	8	9	10	Analysis Objects*^1^
1	CR	0.328	0.285	0.195	0.075	0.054	0.036	0.018	0.009	0.000	0.000	3
	CCR	0.328	0.613	0.883	0.937	0.973	0.991	1.000	1.000	1.000	1.000	
2	CR	0.426	0.179	0.144	0.073	0.052	0.049	0.039	0.023	0.008	0.007	3
	CCR	0.426	0.605	0.749	0.822	0.874	0.923	0.962	0.985	0.993	1.000	

*Note*. CR: contribution rate, CCR: cumulative contribution rate. *^1^ Up to principal components with a CR of at least 0.10 and a CCR of at least 0.700.

In Group 1, the CRs were 0.328, 0.285, 0.195, 0.075, 0.052, 0.036, 0.0018, 0.009, and 0.000 from the first principal component. The analysis was conducted up to the third principal component, and the CCR was 0.883. In Group 2, the CRs were 0.426, 0.179, 0.144, 0.073, 0.052, 0.049, 0.039, 0.023, 0.008, and 0.007 from the first principal component. The third principal component was examined; the CCR was 0.749.

**Fig 18:**
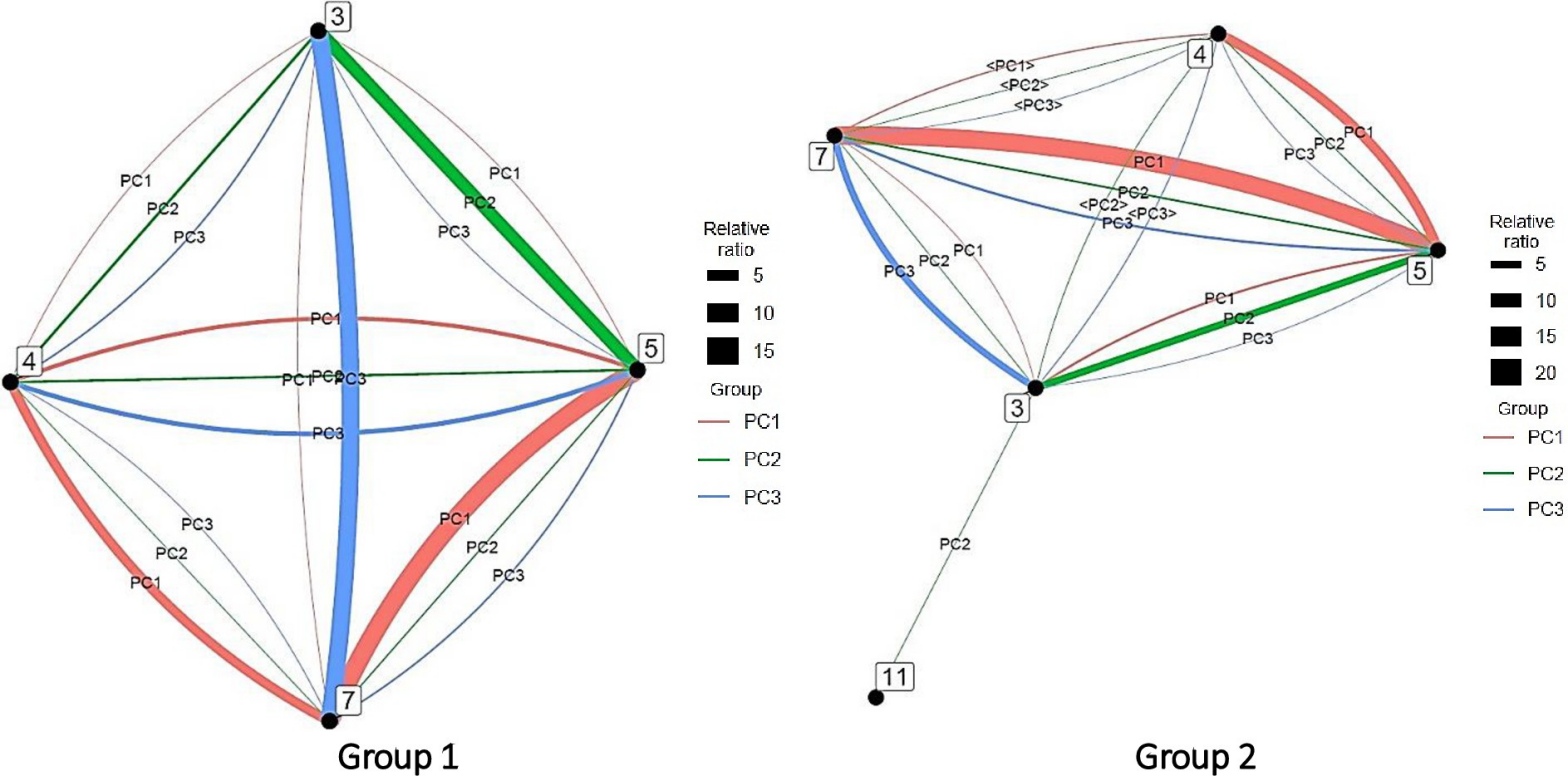
Principal component analysis of each internet of things group. *Note*. <PC#> has factor loadings below 0.300 but are shown as reference information.

The characteristics of each principal component for each group are presented to show the tendency of the connection between fields within the group. The analysis method was similar to that for AI. Fig 18 shows the PCA results for IoT.

In Group 1, the first principal component was indicated by the interdisciplinary connections between computer science and engineering (5–7) and clinical medicine and engineering (4–7); the second main component was characterized by that between chemistry and computer science (3–5); and the third main component was depicted by that between chemistry and engineering (3–7).

In Group 2, the first principal component was characterized by interdisciplinary links between computer science and engineering (5–7) and clinical medicine and computer science (4–5); the second main component was illustrated by that between chemistry and computer science (3–5); and the third main component was indicated by that between chemistry and engineering (3–7).

### Summary of individual technologies in Industry 4.0 and its relationship

This section presents a cross-country comparison of the links among the top 20 author research areas in AI (four groups), big data (three groups), and IoT (two groups). However, as the differences between the two groups for IoT were small, we considered the top 20 IoT countries as one group. The characteristic of IoT is that chemistry, clinical medicine, computer science, and engineering (3–4–5–7) were complete networks connecting all research fields. Table 6 shows the characteristics of each AI and big data group.

**Table 6:** Characteristics of each artificial intelligence and big data group.

Gr.	AI	Big Data
1	There are strong interdisciplinary links between engineering and environment/ecology (7–8) and environment/ecology and geosciences (8–9). Chemistry, engineering, and environment/ecology (3–7–8) are fully networked. The analysis object is one.	Computer science (5), computer science and engineering (5–7), clinical medicine and computer science (4–5), and chemistry and computer science (3–5). The analysis objects are two.
2	There are strong interdisciplinary links between clinical medicine and neuroscience and behavior (4–16). There are strong interdisciplinary links between clinical medicine (4), chemistry (3), computer science (5), engineering (7), molecular biology and genetics (14), and psychology/psychiatry (20). The analysis object is one.	There are several links, mainly in clinical medicine (4). As there are no notable strong links, it is likely that numerous links exist. The analysis objects are three.
3	Chemistry, clinical medicine, computer science, and engineering (3–4–5–7) are fully networked. The analysis objects are three.	Chemistry, clinical medicine, computer science, and engineering (3–4–5–7) have a complete network. The analysis objects are three.
4	Clinical medicine, computer science, and engineering (4–5–7) have complete networks. Centered on clinical medicine (4), interdisciplinary links exist between biology and biochemistry (2), molecular biology and genetics (14), and multidisciplinary (15). The analysis objects are three.	NA

In the top 20 AI countries, in Group 1, the interdisciplinary connections are strong between engineering and environment/ecology (7–8) and environment/ecology and geosciences (8–9). Additionally, chemistry, engineering, and environment/ecology (3–7–8) have a complete network linking all research fields. The analysis object of Group 1 is one. In Group 2, clinical medicine and neuroscience and behavior (4–16) have strong interdisciplinary connections. The analysis object of Group 2 is one. Further, Group 3 is characterized by strong interdisciplinary connections among clinical medicine (4), chemistry (3), computer science (5), engineering (7), molecular biology and genetics (14), and psychology/psychiatry (20), with the first four constituting a complete network connecting all research fields (3–4–5–7). The analysis objects of Group 3 are three. Finally, in Group 4, clinical medicine, computer science, and engineering (4–5–7) are a complete network connecting all research fields, and clinical medicine (4), biology and biochemistry (2), molecular biology and genetics (14), and multidisciplinary (15) are connected to one another. The analysis objects of Group 4 are three.

The top 20 big data countries were classified into three groups for the ease of interpretation: Group 1 consisted of Saudi Arabia, Pakistan, Iran, and Malaysia. Group 2 included France, Germany, the Netherlands, Canada, the United States, England, Spain, Australia, and Italy. Group 3 was composed of Brazil, Taiwan, Japan, India, Singapore, China, and South Korea.

In the top 20 big data countries, in Group 1, we found that the center was computer science (5), followed by computer science and engineering (5–7), clinical medicine and computer science (4–5), and chemistry and computer science (3–5). The analysis objects of Group 1 are two. Group 2 showed many connections, especially in clinical medicine (4). Additionally, as there were no exceptionally strong connections, we can assume that a large number of broad ones exist. The analysis objects of Group 2 are three. Finally, in Group 3, chemistry, clinical medicine, computer science, and engineering (3–4–5–7) have a complete network connecting all research fields. The analysis objects of Group 3 are three.

#### Relationship between each technology in Industry 4.0

**Fig 19:**
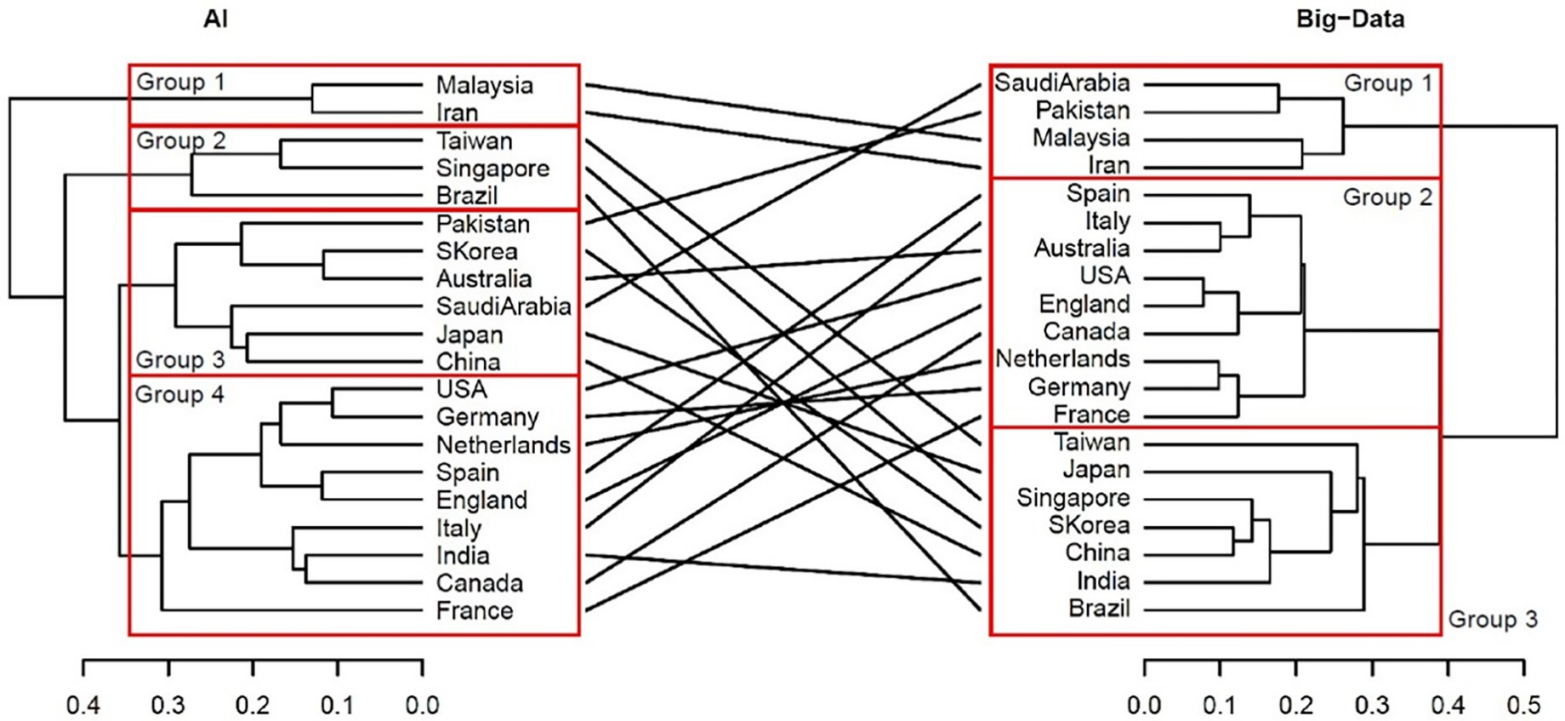
Country similarity tanglegram: Links between author research areas in the top 20 countries for artificial intelligence and big data. *Note*. Distance: hclust (*, “ward.D2”).

Based on AI and big data trends, we developed a country-by-country comparison of the links between the author research fields of the top 20 countries in Industry 4.0. Fig 19 shows the similarity tanglegram among the top 20 AI and big data countries in terms of the links between author research fields. Some dominant linkages are shown in the intergroup relationships among the top 20 AI and big data countries: Group 1 in both AI and Big data, Group 2 in AI and Group 3 in Big data, Group 3 in both AI and Big data, and Group 4 in AI and Group 2. Other connections are also recognized.

Therefore, Table 7 shows the seven connection patterns between the author research areas identified in the top 20 AI and big data fields. The author research areas are grouped into seven groups. The groups from Malaysia and Iran were in Group 1 for both AI and Big data. Taiwan, Singapore, and Brazil groups were in Group 2 for AI and Group 3 for big data. The Middle East, Pakistan, and Saudi Arabia were in Group 3 for AI and Group 4 for Big data. East Asia, China, Japan, and South Korea were in Group 3 for both AI and Big data. The United States, Germany, Netherlands, Spain, England, Italy, Canada, and France in Western countries were in Group 4 for AI and Group 2 for big data. Australia alone was in Group 3 for AI and Group 2 for Big data. India was alone in Group 4 for AI and Group 3 for Big data.

**Table 7:** Seven connection patterns between the author research areas.

#	Countries or Regions	Group of Research Areas	
		AI	Big Data
1	Malaysia, Iran	1	1
2	Taiwan, Singapore, Brazil	2	3
3	Pakistan, Saudi Arabia	3	1
4	China, Japan, South Korea	3	3
5	USA, Germany, Netherlands, Spain, England, Italy, Canada, France	4	2
6	Australia	3	2
7	India	4	3

## Discussion and conclusions

This study contributes to the development of Industry 4.0 by identifying cross-disciplinary fusion patterns in AI, big data, and IoT based on Innovation theory.

Innovation is a thinking approach that creates new knowledge (value) from “new combinations of knowledge, resources, and experience in economic activities,” which Schumpeter [18] called new combination in business administration. This study uses the definition of existing knowledge as an interdisciplinary field and considers new knowledge (value) created by fusion of these disciplines as innovation. This study examines how each country is promoting research from the perspective of innovation. The analysis method includes measuring the "intrapersonal diversity" of Schumpeterian competition, which is an innovation strategy in Barney’s three major types of interorganizational competition [6]. The competitiveness of an organization (in this study, a country/region) is evaluated by accumulating the intrapersonal diversity of its members. For example, in Region A, if the disciplines of clinical medicine and computer science are strongly connected (many researchers are involved in research in both disciplines), the knowledge of these disciplines will be combined. In this case, it is likely that new value (knowledge) is created by fusing the knowledge of these disciplines.

The reader may wonder why this study focuses on the development of each field instead of innovation-type development. These are the ideas of IO-type and Chamberlain-type competition in Barney’s three major interorganizational competitions [6]. However, both are suited to industries where the business environment is fairly stable and the future is reasonably foreseeable. However, the current business environment may be different. Globalization, deregulation, and, above all, rapid development and digitization of IT have accelerated change in the business environment. D’aveni [19] refers to this environment as "hypercompetition," meaning that the type of competition is adapting to the Schumpeterian model. This study attempts to gain knowledge on competitive strategies that are appropriate for this hypercompetitive economic situation.

This study categorized the styles of cross-disciplinary fusion into four patterns in AI and three patterns in big data. In IoT, the results showed only small differences between countries, so this study did not discuss them.

There were regional differences in the style of cross-disciplinary fusion in AI and big data. In Europe and North America, the style was similar between the United States, Germany, the Netherlands, Spain, England, Italy, Canada, and France. In AI, clinical medicine, computer science, and engineering (4–5–7) were fully networked in these countries, showing the central feature of cross-disciplinary integration. Interdisciplinary links were also found between clinical medicine (4), biology and biochemistry (2), molecular biology and genetics (14), and multidisciplinary (15). Several links were found in big data, mainly in clinical medicine (4). Furthermore, as there were no outstandingly strong links, many broad links can be assumed.

Additionally, the cross-disciplinary fusion style was similar between China, Japan, and South Korea in Asia. In AI, chemistry, clinical medicine, computer science, and engineering (3–4–5–7) formed a complete network in these countries. Hence, these countries have an advanced cross-disciplinary integration in chemistry, which was not the case in Europe and North America. In big data, chemistry, clinical medicine, computer science, and engineering (3–4–5–7) formed a complete network as well. Other countries with a similar style of cross-disciplinary integration were Malaysia, Iran, Taiwan, Singapore, Brazil, Pakistan, and Saudi Arabia, while Australia and India showed unique styles.

Furthermore, this study showed that regional differences exist in interdisciplinary fusion styles. This may be outside the scope of this study, or it may be related to "Exploitation of knowledge." March [5] stated that "Exploitation of knowledge" in the innovation process is an activity that deepens knowledge by combining nearby knowledge "already known" and a process that allows organizations to generate revenue. Alternatively, it can be interpreted that the regional nature of the interdisciplinary fusion style is related to proximity, such as geographical distance or economic dependence. Further research is needed to verify this phenomenon.

The reader may be interested to know whether the effects of the linkage between the various fields revealed in this study are actually manifested in actual results. This point should be verified through empirical research. However, in the case of social science, to measure the effect of a theory, it is necessary to assume that it will take several years for the effect to spread in the relevant industry.

A future direction would be to conduct empirical research to verify the effectiveness of the graphs of organizational research capacity and interdisciplinary connections created in this study, as well as the graphs of interdisciplinary connections that characterize each group, from the perspective of Porter’s [17] activity system. Although the activity system is positioned as a practical framework for realizing Dierickx and Cool’s [16] "resource imitation difficulty," it can be used to verify the effectiveness of this research.

In the person data in this study, the only separation of multiple persons with the same name is by country/region. As this study considers trends by country, it is assumed that some confusion of the same name occurs in each country/region. In this study, we classify each author based on WoS categories, but this classification is not derived from papers but from whole journals. These are the limitations of this study.
